# Multi‐omics analysis reveals comprehensive aberrant protein and phosphorylation characteristics in breast cancer and paired metastatic lymph nodes

**DOI:** 10.1093/procel/pwag002

**Published:** 2026-01-12

**Authors:** Linhui Zhai, Cui-Cui Liu, Lei Zhao, Le-Wei Zheng, Chengyu Chu, Hong Hu, Yu-Wen Cai, Lie Chen, Yi-Ming Liu, Yiou Wang, Wensi Zhao, Yuqi Huang, Shiyu Duan, Zhi-Ming Shao, Yiting Jin, Minjia Tan, Ke-Da Yu

**Affiliations:** Department of Breast Surgery, Key Laboratory of Breast Cancer in Shanghai, Fudan University Shanghai Cancer Center and Cancer Institute, Department of Oncology, Shanghai Medical College, Fudan University, Shanghai 200032, China; Translational Research Institute of Brain and Brain-Like Intelligence, Shanghai Fourth People’s Hospital, and Cancer Center, School of Medicine, Tongji University, Shanghai 200434, China; State Key Laboratory of Drug Research, Shanghai Institute of Materia Medica, Chinese Academy of Sciences, Shanghai 201203, China; Department of Breast Surgery, Key Laboratory of Breast Cancer in Shanghai, Fudan University Shanghai Cancer Center and Cancer Institute, Department of Oncology, Shanghai Medical College, Fudan University, Shanghai 200032, China; Translational Research Institute of Brain and Brain-Like Intelligence, Shanghai Fourth People’s Hospital, and Cancer Center, School of Medicine, Tongji University, Shanghai 200434, China; Department of Thyroid and Breast Surgery, General Surgery, Huashan Hospital, Fudan University, Shanghai 200040, China; Zhongshan Institute for Drug Discovery, Shanghai Institute of Materia Medica, Chinese Academy of Sciences, Zhongshan, 528400, China; Department of Breast Surgery, Key Laboratory of Breast Cancer in Shanghai, Fudan University Shanghai Cancer Center and Cancer Institute, Department of Oncology, Shanghai Medical College, Fudan University, Shanghai 200032, China; Shanghai Institute of Infectious Disease and Biosecurity, Fudan University, Shanghai 200040, China; Department of Thyroid and Breast Surgery, General Surgery, Huashan Hospital, Fudan University, Shanghai 200040, China; Division of Breast Surgery, Department of General Surgery, Shenzhen People’s Hospital, The Second Clinical Medical College, Jinan University; The First Affiliated Hospital, Southern University of Science and Technology, Shenzhen 518020, China; Department of Breast Surgery, Key Laboratory of Breast Cancer in Shanghai, Fudan University Shanghai Cancer Center and Cancer Institute, Department of Oncology, Shanghai Medical College, Fudan University, Shanghai 200032, China; Department of Breast Surgery, Key Laboratory of Breast Cancer in Shanghai, Fudan University Shanghai Cancer Center and Cancer Institute, Department of Oncology, Shanghai Medical College, Fudan University, Shanghai 200032, China; Department of Breast Surgery, Key Laboratory of Breast Cancer in Shanghai, Fudan University Shanghai Cancer Center and Cancer Institute, Department of Oncology, Shanghai Medical College, Fudan University, Shanghai 200032, China; Department of Breast Surgery, Key Laboratory of Breast Cancer in Shanghai, Fudan University Shanghai Cancer Center and Cancer Institute, Department of Oncology, Shanghai Medical College, Fudan University, Shanghai 200032, China; Department of Thoracic Surgery, Shanghai Pulmonary Hospital, School of Medicine, Tongji University, Shanghai 200433, China; Department of Thyroid and Breast Surgery, General Surgery, Huashan Hospital, Fudan University, Shanghai 200040, China; Department of Thyroid and Breast Surgery, General Surgery, Huashan Hospital, Fudan University, Shanghai 200040, China; Department of Breast Surgery, Key Laboratory of Breast Cancer in Shanghai, Fudan University Shanghai Cancer Center and Cancer Institute, Department of Oncology, Shanghai Medical College, Fudan University, Shanghai 200032, China; Department of Thyroid and Breast Surgery, General Surgery, Huashan Hospital, Fudan University, Shanghai 200040, China; State Key Laboratory of Drug Research, Shanghai Institute of Materia Medica, Chinese Academy of Sciences, Shanghai 201203, China; Department of Thyroid and Breast Surgery, General Surgery, Huashan Hospital, Fudan University, Shanghai 200040, China; Zhongshan Institute for Drug Discovery, Shanghai Institute of Materia Medica, Chinese Academy of Sciences, Zhongshan, 528400, China; Department of Breast Surgery, Key Laboratory of Breast Cancer in Shanghai, Fudan University Shanghai Cancer Center and Cancer Institute, Department of Oncology, Shanghai Medical College, Fudan University, Shanghai 200032, China; Shanghai Institute of Infectious Disease and Biosecurity, Fudan University, Shanghai 200040, China

**Keywords:** breast cancer, early-stage metastasis, proteogenomic, alternative splicing, MARCKSL1-S104, immune escape

## Abstract

Breast cancer is the most frequently diagnosed cancer, with metastasis accounting for the majority of cancer-related deaths. The mechanisms of early-stage breast cancer metastasis to regional immune sites like lymph nodes remain elusive. Here, we performed an in-depth proteomic and phosphoproteomic analyses of a substantial series of breast cancer samples, alongside genomic and transcriptomic evaluations. This cohort encompasses 195 specimens: 65 primary breast tumors, their corresponding normal tissues, and metastatic axillary lymph nodes. We offer an overview of the molecular alterations at the transcriptomic, proteomic, and phosphoproteomic levels during lymph node metastasis. Notably, the findings indicate that regional lymph node metastasis is primarily influenced by proteomic and phosphoproteomic alterations, rather than genomic or transcriptomic changes. We found that ANGPTL4 and HMGB1 could serve as biomarkers of lymph node metastasis. Data analysis and cell experiments involving silencing of the alternative splicing (AS) factor HNRNPU demonstrated that AS plays a significant role in modulating protein expression, phosphorylation profiles, and cell proliferation. The key phosphorylation sites, including MARCKSL1-S104 and FKBP15-S320, as well as the upstream kinase PRKCB, were identified as playing crucial roles in breast cancer lymph node metastasis. Targeted intervention of the kinase PRKCB resulted in effectively suppressing the proliferation and metastasis of breast cancer tumor cells. Immune profiling analysis and experimental validation of breast cancer cell co-cultured with CD8^+^ T cell reveals correlations between phosphorylation of MARCKSL1-S104 and FKBP15-S320 with immune checkpoint PD-L1 expression, and their impact on tumor cell apoptosis, suggesting a potential mechanism of immune evasion in metastasis. This study systematically characterizes the molecular landscape and features of primary breast tumors and their matched metastatic lymph nodes. These insights enhance our understanding of early-stage breast cancer metastasis and may pave the way for improved diagnostic tools and targeted therapeutic strategies.

## Introduction

Breast cancer, characterized by rapid proliferation, treatment resistance, and frequent metastasis, accounts for nearly 30% of all female cancers worldwide (Bray et al., 2024; Siegel et al., 2024). Metastasis remains the primary cause of mortality in breast cancer patients, with a 5-year relative survival rate of around 20%–40%, contributing to the majority of cancer-related deaths (Giaquinto et al., 2022; Siegel et al., 2024; Sundquist et al., 2017). Lymph node metastasis is a critical early step in the progression to distant organs and is associated with decreased survival rates (Hyman et al., 2017; Liu et al., 2023b). Despite its significance, the biological mechanisms and multi-omics-level molecular characteristics associated with lymph node metastasis remain poorly understood, highlighting the urgent need for in-depth investigation into these molecular features and their related pathways.

Current genomic-based therapies face limitations in precision medicine, as some mutations do not result in detectable changes at the protein level (Ginsburg et al., 2017; Reticker-Flynn et al., 2022; Ventre et al., 2022). Therefore, a comprehensive approach to identify potential therapeutic targets is essential (Cancer Genome Atlas, 2012; Mertins et al., 2016; Perou et al., 2000). Multi-omics studies, including proteomics and phosphoproteomics, offer a more detailed understanding of the complex cellular behaviors driving disease onset, progression, and metastasis, particularly in highly heterogeneous disorders (Chen et al., 2023a; Jiang et al., 2019; Liu et al., 2023a, 2024; Petralia et al., 2024). These strategies provide novel insights into cancer mechanisms, diagnosis, and prognosis prediction, thereby advancing the field of cancer research (Curtis et al., 2012).

Although omics studies have been widely applied to various cancers, most have focused on primary tumors (Coates et al., 2015; Perou et al., 2000). While several studies have conducted on genomics and transcriptomics in metastatic breast cancer, these approaches often failed to capture the complexity between primary and metastatic tumors (Condorelli et al., 2019; Pan et al., 2020). Consequently, the protein landscape alterations associated with lymph node metastasis in breast cancer, including key biological pathways and critical post-translational modification (PTM) events, remain insufficiently characterized. This emphasizes the necessity of comprehensive multi-omics analyses utilizing paired tissue samples to gain deeper insights into the molecular mechanisms underlying metastasis.

This study employed a multi-omics approach to comprehensively characterize molecular alterations across multiple omics levels in primary breast tumors and metastatic axillary lymph nodes. The findings revealed that proteomic and phosphoproteomic changes, rather than genomic or transcriptomic, predominantly deriven breast cancer lymph node metastasis. Data analysis and cell experiments involving silencing of the alternative splicing (AS) factor HNRNPU demonstrated that AS played a key role in modulation of protein and phosphorylation profiles, as well as cell proliferation. The key phosphorylation sites, including MARCKSL1-S104 and FKBP15-S320, were identified as playing crucial roles in breast cancer lymph node metastasis. Targeted inhibition of the upstream kinase PRKCB was demonstrated to effectively suppress the proliferation and metastasis of breast cancer tumor cells. Immune profiling analysis and experimental validation of breast cancer cells co-cultured with CD8^+^ T cell revealed correlations between phosphorylated proteins and immune checkpoint expression, as well as their impact on tumor cell apoptosis, suggesting a potential mechanism of immune evasion in metastasis. The study provides a comprehensive data resource that enhances the understanding of early-stage breast cancer metastasis, supports the identification of potential biomarkers, and informs the development of potential diagnostic and therapeutic strategies.

## Results

### Integrated multi-omics analysis of early-stage breast cancer metastasis 

**Figure 1.**
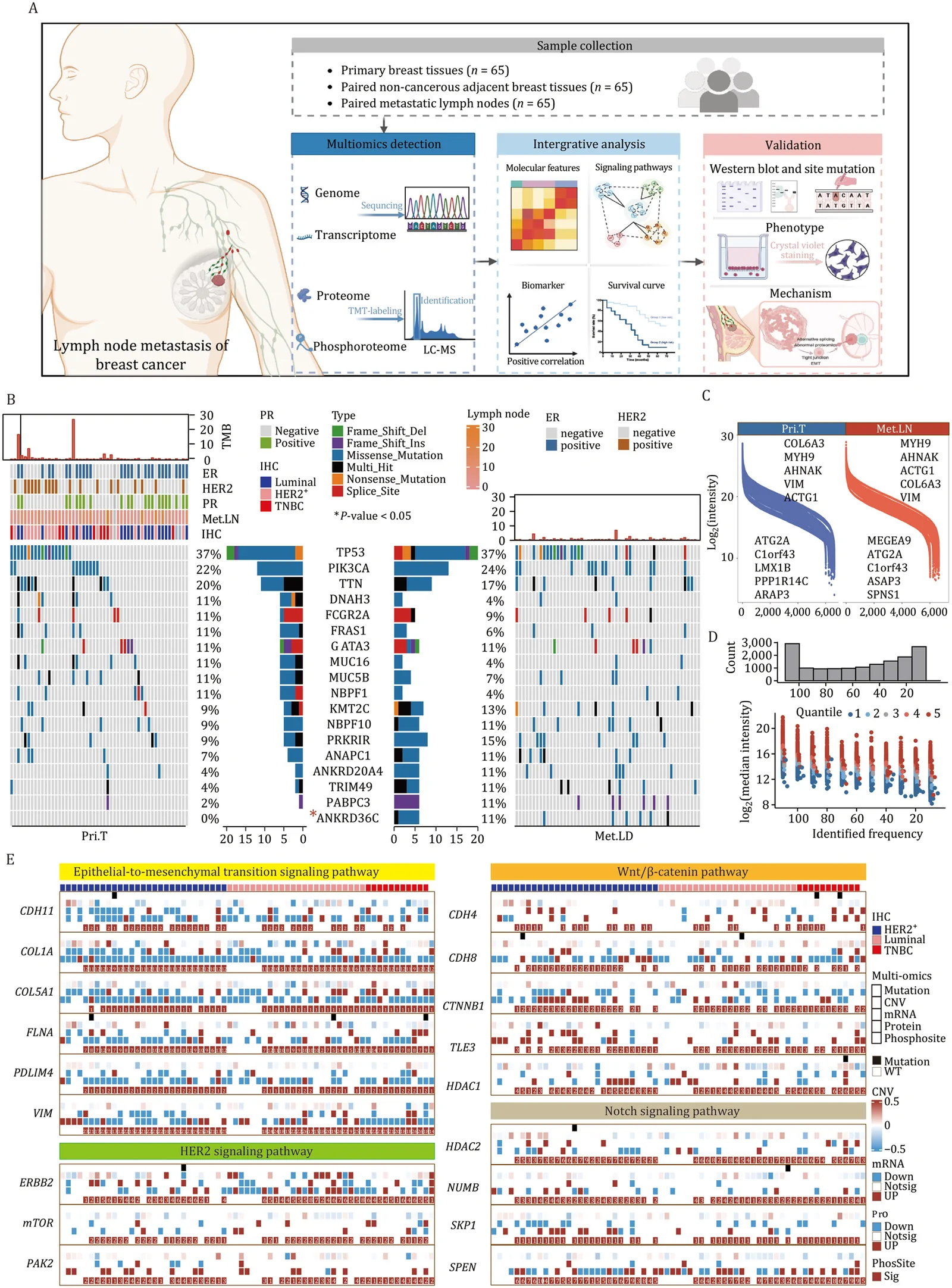
**Integrated multi-omics analysis of early-stage metastasis of breast cancer**. (A) Comprehensive proteogenomic workflow detailing NATs, Pri.Ts, and Met.LNs analysis, including sample collection, proteogenomic detection, integrative analysis, and biological validation. A total of 195 tissue samples were collected, including 65 paired primary tumors (Pri.Ts), metastatic lymph nodes (Met.LNs), and normal adjacent tissues (NATs) from surgical resection. (B) Comparative analysis of genes with high-frequency mutations occurring in a substantial proportion of the population (>10%, *n* > 6) between Pri.Ts and Met.LNs. *represented fisher’s exact test *P* < 0.05. (C) Overview of proteomic profiles in Pri.Ts and Met.LNs. Protein abundance dynamics are shown in blue for tumors and red for lymph nodes, with the highest and lowest abundance proteins listed. Abundance is quantified using log_2_ transformed TMT labeling intensity values. (D) Distribution of log_2_ transformed median intensities for phosphorylation sites with varying identification frequencies across all tissue samples. The bar plot shows cumulative counts of identified phosphorylation sites. (E) Heat map displaying gene mutations, CNV changes, transcription levels, altered protein expression, and number of altered phosphorylation sites for key proteins implicated in pathways closely related to breast cancer metastasis across paired Pri.Ts and Met.LNs. Samples are grouped by IHC grade. Differential proteins and phosphosites were filtered for fold-change ratios >1.2 between Pri.T and paired Met.LNs, with an adjusted *P* ≤ 0.05.

To characterize the proteogenomic profiling of early-stage breast cancer metastasis, 195 tissue samples from surgical resection were collected prospectively under standardized protocols from Fudan University Shanghai Cancer Center (FUSCC), including 65 paired treatment-naïve primary tumors (Pri.Ts), metastatic lymph nodes (Met.LNs), and normal adjacent tissues (NATs). The clinicopathological characteristics are summarized in Table S1A and Fig. S1A. A schematic of the experimental design is shown in Fig. 1A. All samples were cryo-pulverized and aliquoted for molecular profiling using whole-exome sequencing (WES), RNA sequencing (RNA-seq), and isobaric tandem mass tag (TMT) labeling-based global proteomics and phosphoproteomics (Fig. S1B; Table S1B). Before conducting multi-omics analysis, we ensured the quality of genomic DNA, RNA, and proteins by excluding samples with signs of degradation. Ultimately, 54 Pri.T and 58 Met.LN samples underwent whole-exome sequencing (WES); 51 NAT samples, 55 Pri.T samples, and 58 Met.LN samples underwent RNA-seq; 65 Pri.T and 65 Met.LN samples were profiled for protein expression; and 65 Pri.T and 55 Met.LN samples were analyzed for phosphoproteome profiling. The distribution of subtype frequencies in the final enrolled cohort across omics layers is summarized in Table S1C and S1D, demonstrating a balanced representation of subtypes and indicating no significant bias in subtype sampling in this study. In total, 7,280 nonsynonymous somatic mutations and 25,988 somatic copy-number variations (SCNVs) were identified (Tables S2 and S3A), with minimal difference between Pri.Ts and Met.LNs (Figs. 1B and S1C–E). RNA-seq analysis quantified 16,892 genes with fragments per kilobase of transcript per million fragments mapped (FPKM) above 0, facilitating the exploration of the relationship between the transcriptome and full proteome (Fig. S1F; Table S3B). TMT-based proteomic analysis identified 8,588 proteins (Figs. 1C and S1G; Table S3C), while Ti^4+^-IMAC-based phosphoproteomic analysis identified 24,178 confidently localized phosphosites from 5,182 phosphoproteins (Figs. 1D and S1H; Table S3D). High reproducibility, technical quality, and the absence of batch effects were demonstrated across all TMT-labeling groups, including both proteomics and phosphoproteomics datasets (Fig. S1I–L). Collectively, our study provides high-quality, multi-dimensional data that significantly support research on breast cancer lymph node metastasis.

Based on WES results, it can be speculated that since tumor tissue and metastatic lymph node tissue originate from the same patient, the number of gene mutations occurring before and after metastasis may be relatively limited. The correlation between overall protein and mRNA expression levels as Pri.Ts and Met.LNs using Spearman’s correlation, revealing a significant correlation (Spearman correlation, rho = 0.4, *P* < 0.0001) (Fig. S1M). At the sample-wise level, Met.LNs exhibited a median sample-wise correlation coefficient of 0.39, while Pri.Ts showed a median correlation coefficient of 0.40 (Fig. S1N). These results provide valuable insights into the molecular similarities and distinctions between Met.LNs and their corresponding Pri.Ts. Meanwhile, the relatively modest difference in mRNA-protein correlations observed between metastatic Met.LNs and Pri.Ts indicates the distinct characteristics at the transcriptomic and proteomic levels.

The metastasis of breast tumors has been widely reported to be closely associated with the regulation of multiple signaling pathways. We subsequently analyzed the differences in gene mutations, transcription level, protein expression, and phosphorylation modification levels of genes within pathways–specifically EMT, Wnt/β-catenin, HER2, and Notch signaling pathway—that are closely associated with breast cancer metastasis between Pri.Ts and Met.LNs. Representative genes from each pathway were selected for detailed presentation and analysis. Although genetic mutations are detectable, they occur at relatively low frequencies. Most genes display differential changes primarily at the copy number variation (CNV) and transcriptional levels. However, changes at the proteomic and phosphoproteomic levels are more pronounced compared to those observed at the genomic and transcriptional levels (Fig. 1E). Overall, this study has established a comprehensive proteogenomic landscape and provided an in-depth data resource for understanding breast cancer lymph node metastasis across genomic, transcriptomic, proteomic, and phosphoproteomic levels.

### Proteomic alteration and potential biomarkers in lymph nodes metastasis

The differences in gene expression levels within specific pathways across various molecular levels highlight the significance of alterations in protein and phosphorylation levels. Accordingly, we conducted comprehensive multi-dimensional comparative analyses to further investigate these variations. We conducted a mutation analysis by comparing tumor and lymph node samples using WES data. A complete list of all identified mutated genes is provided in Table S2. Considering the sample size of the Pri.Ts and Met.LNs, and to ensure robust statistical power, we focused exclusively on genes with high-frequency mutations occurring in a substantial proportion of the population (>10%, *n* > 6). Consequently, the top 18 genes with the highest mutation frequencies were selected for further analysis (Figs. 1B and S1C). In the FUSCC cohort analyzed in this study, the most frequently mutated genes were *TP53*, *PIK3CA*, and *TTN*, with mutation rates of 37%, 22%, and 20% in Pri.Ts, and 37%, 24%, and 17% in Met.LNs, respectively. Consistent with previous study, *P53* and *PIK3CA* have been identified as the most frequently mutated genes in Chinese breast cancer patients (Lang et al., 2020). We further compared the gene mutation frequencies in both the TCGA dataset and our in-house dataset. As shown in the subsequent figure, there is a significant consistency between these two datasets (Fig. S1E). Notably, additional mutations with frequencies greater than 10% were identified in genes encoding chromatin-modifying enzymes (*KMT2C*, *ANAPC1*), microtubule-associated motor proteins (*DNAH3*, *FRAS1*), transcriptional regulators (*GATA3*, *NBPF1*, *NBPF10*), and members of the *ANKRD* family (*ANKRD20A*, *ANKRD36C*). Most of these genes have also been reported to be mutated in breast cancer. Through integrative analyses of somatic gene mutations, only *ANKRD36C* exhibited significant alterations in Met.LNs (11%) compared to Pri.Ts (0%) at the genomic level (Fig. 1B). The results indicate that the differences in gene mutations between Met.LNs and Pri.Ts are relatively minor, suggesting that breast cancer lymph node metastasis may be less associated with genetic mutations.

**Figure 2.**
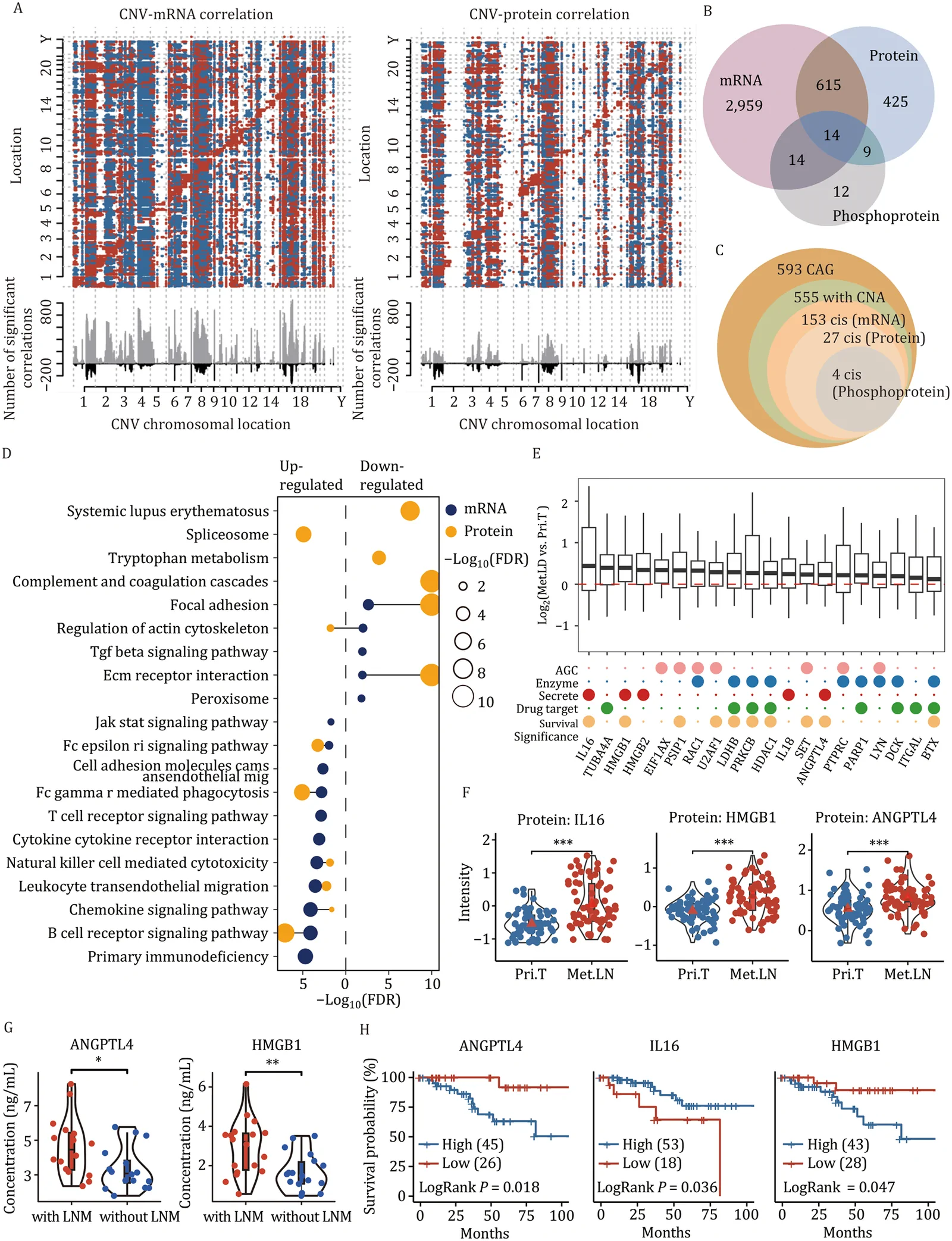
**Proteomic alterations and potential biomarkers in lymph nodes metastasis**. (A) Impact of cis (diagonal lines) and trans effects of CNAs on mRNA and protein levels using all Pri.Ts or Met.LNs samples. Genes are sorted by chromosome location. The top panel shows correlations between CNAs and mRNA or protein abundance, with significant positive correlations in red and negative correlations in blue (adjusted *P* < 0.01, Spearman’s correlation coefficient). Significant correlations at both mRNA and protein levels for each CNV are labeled in black. (B) Venn diagram analysis showing significant cis effects of Somatic copy number variations (CNVs) using all samples at the transcriptome (*n* = 98), proteome (*n* = 108), and phosphoproteome (*n* = 90) levels (spearman’s *P* ≤ 0.05). (C) Overlap analysis of significant cis events among 593 CAGs using all samples at the transcriptome (*n* = 98), proteome (*n* = 108), and phosphoproteome (*n* = 90) levels. (D) GSEA-based KEGG pathway enrichment analysis of transcriptome and proteome data between Pri.Ts (*n* = 65) and paired Met.LNs (*n* = 65). Significant pathways from transcriptome data are labeled in blue, while significant pathways from proteome data are labeled in yellow (FDR ≤ 0.05). (E) Boxplot showing log2 fold change between Pri.Ts (*n* = 65) and paired Met.LNs (*n* = 65) for cancer-associated proteins (CAG) annotated with potential clinical utilities by the Human Protein Atlas. (F) Comparison of IL16, HMGB1, and ANGP14 protein levels between Pri.Ts (*n* =65) and paired Met.LNs (*n* = 65) (Wilcoxon rank sum and signed rank test). ***P < 0.001. (G) The plasma protein levels of ANGPTL4 and HMGB1 measured by ELISA in an independent cohort consisting of 16 breast cancer patients with lymph node metastasis (with LNM) and 16 non-metastatic breast cancer controls (without LNM). Statistical significance was assessed using the Wilcoxon rank -sum and signed rank test. *P < 0.05 and **P < 0.01. (**H**) Kaplan-Meier curves of disease-free survival based on high or low protein levels of ANGPTL4, IL16, and HMGB1 from 71 TCGA breast cancer samples.

Subsequently, we conducted an analysis of the somatic copy number variations (SCNVs) and identified amplifications in the 17q12 and 1q11 chromosomal regions, as well as deletions in the 6p22.1 and 5q13.2 regions (Fig. S2A). Notably, the most significant amplifications observed in our samples were located in the 17q12 and 1q11 regions. Specifically, the *ERBB2* amplification, which is strongly associated with breast cancer, was detected in the 17q12 region. Integrative multi-omics analysis revealed 138 genes with amplification, of which 57 showed transcriptional evidence and 36 exhibited protein-level expression. However, only 4 out of 31 genes exhibited significant upregulation at protein level in Met.LNs. For gene deletions, 634 out of 3,367 genes were detected at both the transcriptional and protein levels, with 27 proteins showing upregulated expression in Met.LNs compared to Pri.Ts (Fig. S2B). The impacts of SCNAs on mRNA, protein, and phosphoprotein abundance were systematically evaluated for both cis- and trans-effects (Fig. 2A). A total of 3,602, 1,063, and 49 significant positive cis-correlations were observed for mRNA, protein, and phosphoprotein, respectively (Fig. 2B). Among the 762 significant cis-effects shared across all three omics layers, only 45 proteins exhibited differential abundance between Pri.Ts and paired Met.LNs (Table S3). A similar attenuation trend was observed in a previously reported dataset comprising 593 cancer-associated genes (CAGs) (Dong et al., 2024; Gillette et al., 2020) (Fig. 2C). Among them, 555 genes were identified with SCNAs, 153 exhibited cis effects at the transcript level, and 27 demonstrated cis effects at the protein level. Notably, among the 27 proteins, transcription factor YAP1, tumor suppressor gene IRF6, and ubiquitin-specific protease USP4 have been previously reported to be associated with tumor metastasis (Chen et al., 2024; Yanai et al., 2012; Zhang et al., 2012, 2019). These findings demonstrate the consistency of our data with previously reported data, further indicating that gene alterations and mutational profiles have limited influence in early-stage breast cancer metastasis.

A systematic analysis was further conducted to characterize transcriptomic differences between Pri.Ts and Met.LNs. Additionally, the results of these analyses were compared and integrated with the differentially expressed genes identified through proteomics data analysis. Ultimately, for the transcriptome, a total of 1,162 significantly differentially expressed genes were identified (Fig. S2C). For the proteome, 695 proteins were significantly altered between Pri.Ts and paired Met.LNs, including 502 proteins that were downregulated and 193 proteins that were upregulated in Met.LNs compared to Pri.Ts (Fig. S2D; Table S3F). Pathway enrichment analysis was conducted using the Kyoto Encyclopedia of Genes and Genomes (KEGG) database to investigate differentially expressed genes identified from transcriptomic data and the differentially expressed proteins identified from the proteomic data. The results revealed significant enrichment across multiple biological pathways in both datasets, suggesting that the proteome and transcriptome share certain commonalities (Fig. S2E). For example, the ECM-receptor interaction pathway was consistently downregulated at both the transcriptomic and proteomic levels, whereas immune-related pathways, including the B cell receptor signaling pathway and the T cell receptor signaling pathway, were found to be upregulated at both levels. We further performed pathway enrichment analysis separately on differentially expressed genes and proteins. The results consistently revealed significant enrichment in several key pathways, including focal adhesion, regulation of the actin cytoskeleton, ECM-receptor interaction, as well as immune-related pathways such as natural killer cell-mediated cytotoxicity, leukocyte trans-endothelial migration, chemokine signaling pathway, and B cell receptor signaling pathway. A majority of the pathways enriched in the differential transcriptome were also observed at the protein level, with the exception of a few pathways, including peroxisome, JAK-STAT signaling pathway, and T cell receptor signaling pathway. However, at the protein level, there was unique and significant enrichment in the up-regulation of AS pathways, down-regulation of tryptophan metabolism pathways, and down-regulation of complement and coagulation cascades (Fig. 2D). Notably, AS displayed unique characteristics at the protein level compared to the transcriptional level. Differentially expressed proteins were also assessed across various IHC subtypes in Pri.Ts compared to NATs, revealing significantly enhanced estrogen response in Luminal, increased mTORC1 and E2F target signaling pathways in HER2-positive subtypes, and distinct immune features in the TNBC subtype (Fig. S2F), consistent with previous findings. The upregulation of AS was predominantly observed in Luminal and HER2-positive subtypes.

After removing tissue specific proteins, 192 out of 675 upregulated proteins remained significantly elevated in at least 70% of paired Met.LNs compared to Pri.Ts. We then selected paired samples with tumor purity ≥ 0.65, resulting in 19 matched Pri.T/Met.LN pairs for re-analysis. The high-purity subgroup analysis identified 506 significantly differentially expressed proteins, with 493 retained after excluding tissue-specific proteins. Among these differentially expressed proteins, 45 proteins are established FDA-approved drug targets, such as HDAC1, PARP1, and DPP4, which exhibited notable alterations between Pri.Ts and Met.LNs, suggesting their potential as therapeutic targets (Fig. S2G). Additionally, we conducted an in-depth analysis of the characteristics of differentially expressed proteins in Pri.Ts and Met.LNs. Based on multiple criteria, including their association with CAGs, enzymatic functions, secretory properties, potential as drug targets, and significant correlation with prognosis, we screened out 20 key candidate proteins involved in breast cancer metastasis (Fig. 2E). Among these proteins, five secretory proteins were further selected due to their expression levels showing a strong association with patient clinical prognosis. Specifically, IL16, ANGPTL4, and HMGB1 emerged as promising candidates for potential biomarkers in clinical applications (Figs. 2F and S2H). Notably, the protein expression levels of ANGPTL4 and HMGB1 were significantly correlated with the number of lymph node metastases (Fig. S2I). Importantly, the ANGPTL4 protein exhibited significantly higher expression in Met.LNs than in Pri.Ts, whereas no apparent difference was observed at the transcriptional level between these two groups. To validate the accuracy of these protein markers, clinical samples independent of those used in the original proteomics analysis were collected. A total of 32 individuals were enrolled, forming an independent validation cohort consisting of 16 breast cancer patients with lymph node metastasis and 16 non-metastatic controls. Serum levels of ANGPTL4 and HMGB1 were quantitatively assessed using enzyme-linked immunosorbent assays (ELISA) with specific antibodies. The results revealed significantly higher serum concentrations of both proteins in patients with lymph node metastasis compared to the non-metastatic group (Fig. 2G, Wilcoxon rank sum and signed rank tests, *P* < 0.05). These findings reinforced the reliability and potential clinical utility of the identified protein markers. We further utilized the publicly available TCGA proteomic dataset to investigate the prognostic impact of protein expression levels for the identified secreted proteins. The results revealed that high expression of ANGPTL4 and HMGB1 in breast cancer was significantly associated with worse patient survival, while elevated IL16 expression correlated with better prognosis (Kaplan-Meier analysis, log-rank test, *P* < 0.01) (Fig. 2H).

Above all, these discrepancies between transcriptomic and proteomic data highlight the unique regulatory characteristics of breast cancer metastasis, underscoring their significance in the broader biological context. By utilizing proteomic data, we identified some potential critical biomarkers and potential therapeutic targets.

### Phosphoproteomic insights in early-stage breast cancer metastasis

**Figure 3.**
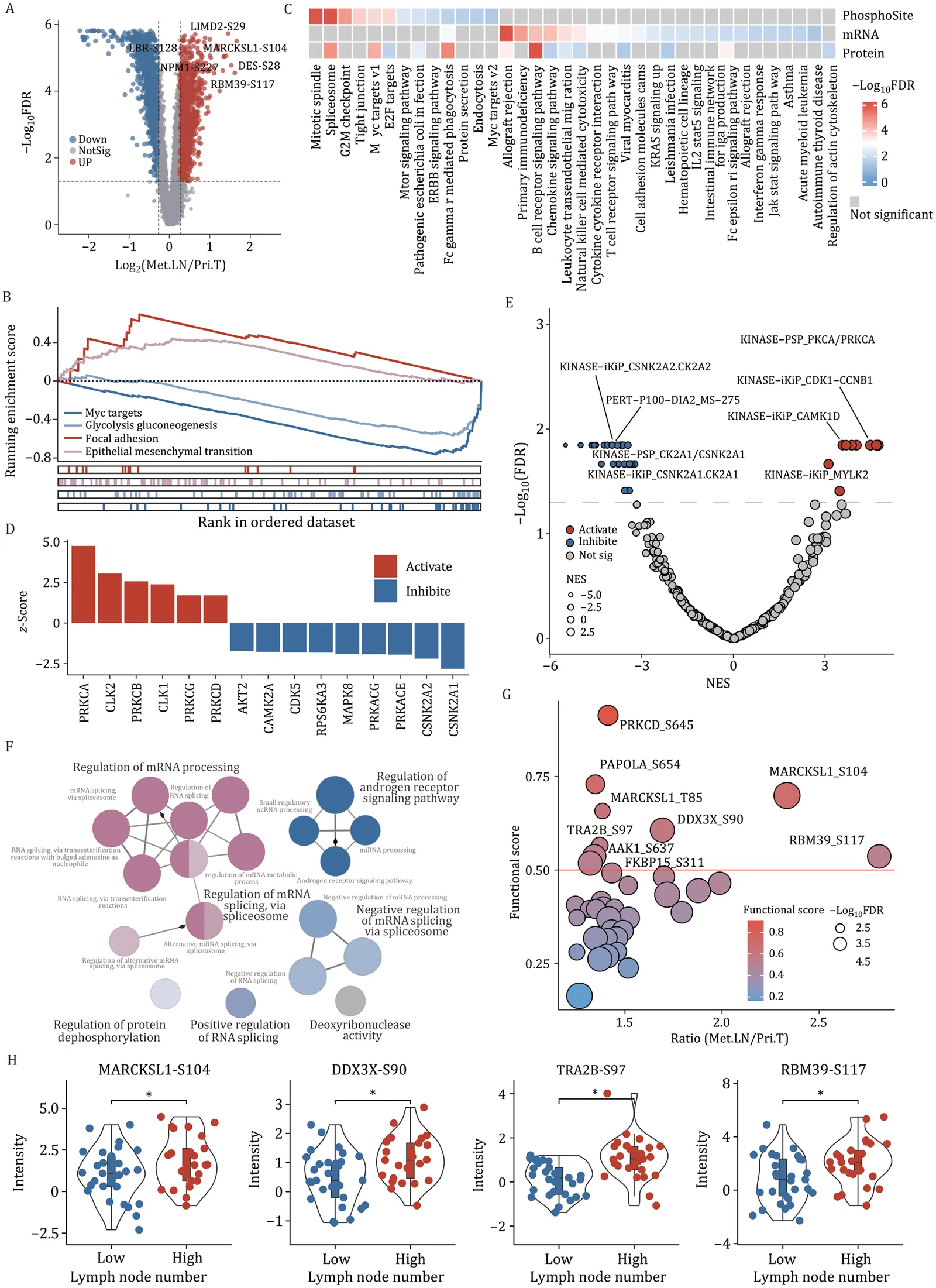
**Phosphoproteomic insights in early-stage breast cancer metastasis**. (A) Differentially expressed phosphorylation sites between Pri.Ts (*n* = 55) and paired Met.LNs (*n* = 55) (adjusted *P*-value from Wilcoxon signed-rank test). Significantly up- or down-regulated phosphorylation sites are marked with red or blue dots, respectively. (B) KEGG pathway enrichment analysis of proteins with upregulated phosphosites in paired Met.LNs (*n* = 55) compared with Pri.Ts (*n* = 55). (C) GSEA-based KEGG pathway enrichment analysis of upregulated phosphosite-related proteins, upregulated proteins, and upregulated genes between Pri.Ts (*n* = 55) and paired Met.LNs (*n* = 55) (FDR ≤ 0.05). (D and E) Significant (FDR < 0.05) kinase signatures between Pri.Ts (*n* = 55) and paired Met.LNs (*n* = 55) assessed by kinase-substrate enrichment analysis (KSEA) and PTM signature enrichment analysis (PTM-SEA). (F) Functional enrichment analysis of phosphorylated proteins corresponding to 62 key phosphorylation sites, revealing significant characteristics associated with alternative splicing. (G) Comparative analysis of expression levels and predicted functional scores for 58 pivotal phosphorylation sites in Pri.Ts (*n* = 55) and corresponding paired Met.LNs (*n* = 55). (H) Differentially expressed pivotal phosphorylation sites between Met.LNs samples with low (*n* ≤ 3) and high (*n* ≥ 4) numbers of lymph nodes metastasis. Wilcoxon rank sum and sign rank test. *P < 0.05.

Phosphorylation is a critical post-translational modification that regulates protein function and intracellular signaling pathways. Given its critical regulatory role, we next investigated its potential role in tumor lymph node metastasis from the perspective of phosphorylation-mediated regulatory mechanisms. A comparative analysis was conducted to examine changes in protein phosphorylation between Pri.Ts and Met.LNs. Principal component analysis (PCA) revealed a clear distinction in phosphorylation levels between Pri.Ts and Met.LNs, with greater heterogeneity observed in Met.LNs (Fig. S3A). Subsequently, a systematic differential analysis of phosphosites and phosphoproteins was performed between Pri.Ts and Met.LNs. After normalizing for total protein abundance, 1,170 upregulated and 991 downregulated phosphosites were identified in Met.LNs compared to Pri.Ts (Fig. 3A). Gene set enrichment analysis (GSEA) of these phosphoproteins with differentially expressed phosphosites indicated upregulation of pathways such as epithelial-mesenchymal transition and Myc targets, alongside downregulation of glycolysis/gluconeogenesis and focal adhesion pathways (Figs. 3B, S3B, and S3C). Furthermore, integrative analysis across transcriptomic, proteomic, and phosphoproteomic data revealed significant involvement of AS at both the protein and phosphorylation levels, with a particularly strong impact on phosphorylation (Figs. 3C and S3D). These results indicated that the participation of phosphorylation in AS plays a crucial role in the early-stage breast cancer metastasis.

Kinase activity was inferred based on the phosphorylation levels of kinase substrates. Kinase-substrate enrichment analysis (KSEA) identified 15 kinases with increased activity in Met.LNs compared to Pri.Ts (Fig. 3D). Among these, six kinases—PRKCA, PRKCB, PRKCD, PRKCG, CLK1, and CLK2 exhibited significant alterations in activity. PTM signature enrichment analysis (PTM-SEA) also revealed potential PKC family kinase activation between Met.LNs and Pri.Ts (Fig. 3E). PTM-SEA also revealed the different alterations by drugs among different subtypes (Fig. S3E). For instance, our results demonstrated that the perturbations effects induced by some chemotherapeutic drugs, such as doxorubicin and cyclosporine, were significantly evident in TNBC samples. These findings indicate that cyclosporine could potentially serve as an efficient therapeutic option for TNBC, which is consistent with previous findings (Abduh, 2023; Sikov et al., 2015).

In order to screen the potential biomarkers between Pri.Ts and paired Met.LNs at both the phosphosite and kinase levels, we conducted a systematic screening of differentially phosphorylated sites between the Pri.Ts and paired Met.LNs samples. After normalizing for total protein expression, we analyzed the phosphorylation profiles across multiple paired Pri.Ts and Met.LNs samples to identify significantly altered phosphorylation events. Considering the potential application of these sites as candidate biomarkers, we required candidate sites to exhibit reproducible differential expression across a large cohort of samples. To enhance specificity, we applied stringent frequency-based filtering criteria: only phosphorylation sites with high expression in at least 60% of the paired Pri.Ts and Met.LNs and low expression in at least 20% of the samples were retained. As a result, we preliminarily identified 190 potential candidate phosphorylation sites. To further improve their detectability and practical applicability as biomarkers, we narrowed the list to include only those sites whose corresponding proteins were either normally expressed or showed an upregulated trend in at least 80% of samples. Ultimately, 62 upregulated phosphosites identified in Met.LNs were selected as final potential biomarkers (Fig. S3F). Pathway enrichment analysis of these associated phosphorylated proteins revealed that they are predominantly involved in RNA regulatory processes and AS (Fig. 3F). Functional prediction scores of these 62 potential candidate phosphosites were further analyzed, leading to the identification of nine significantly upregulated phosphosites with scores exceeding 0.5 in Met.LNs compared to Pri.Ts (Fig. 3G). Among these nine phosphosites, the phosphorylation levels at MARCKSL1-S104, RBM39-S117, DDX3X-S90, and TRA2B-S97 were significantly elevated in lymph nodes with a higher metastatic burden compared to those with fewer metastases (Fig. 3H). According to the KSEA database, we conducted a systematic analysis to identify the upstream kinases regulating these sites and found that they are primarily regulated by kinases belonging to the PKC family (Fig. S3G). This observation aligns well with the overall kinase enrichment analysis of the phosphoproteomics data. Subsequently, we analyzed the protein expression levels and phosphorylation states of these kinases. The results revealed that, compared to Pri.Ts, most of these kinases exhibited more significant alterations in phosphosite abundance relative to their corresponding protein abundance in Met.LNs (Fig. S3H). This finding suggests that these kinases may be activated within the lymph node samples. Collectively, these results demonstrate that the proteome and phosphoproteome exhibit unique features, providing new insights and opportunities for identifying novel functionally important phosphosites.

### Aberrant AS regulated early-stage breast cancer metastasis

AS is a critical process in post-transcriptional mRNA modification, generating various mature mRNAs with distinct structures and functions. Increasing evidence has shown that widespread AS is the primary source of protein diversity in over 90% of human genes, making it a key molecular marker in human cancers and a potential target for novel cancer therapies (Kahles et al., 2018). Our proteogenomic data suggested that AS may have promoted lymph node metastasis in breast cancer, warranting further validation.

**Figure 4.**
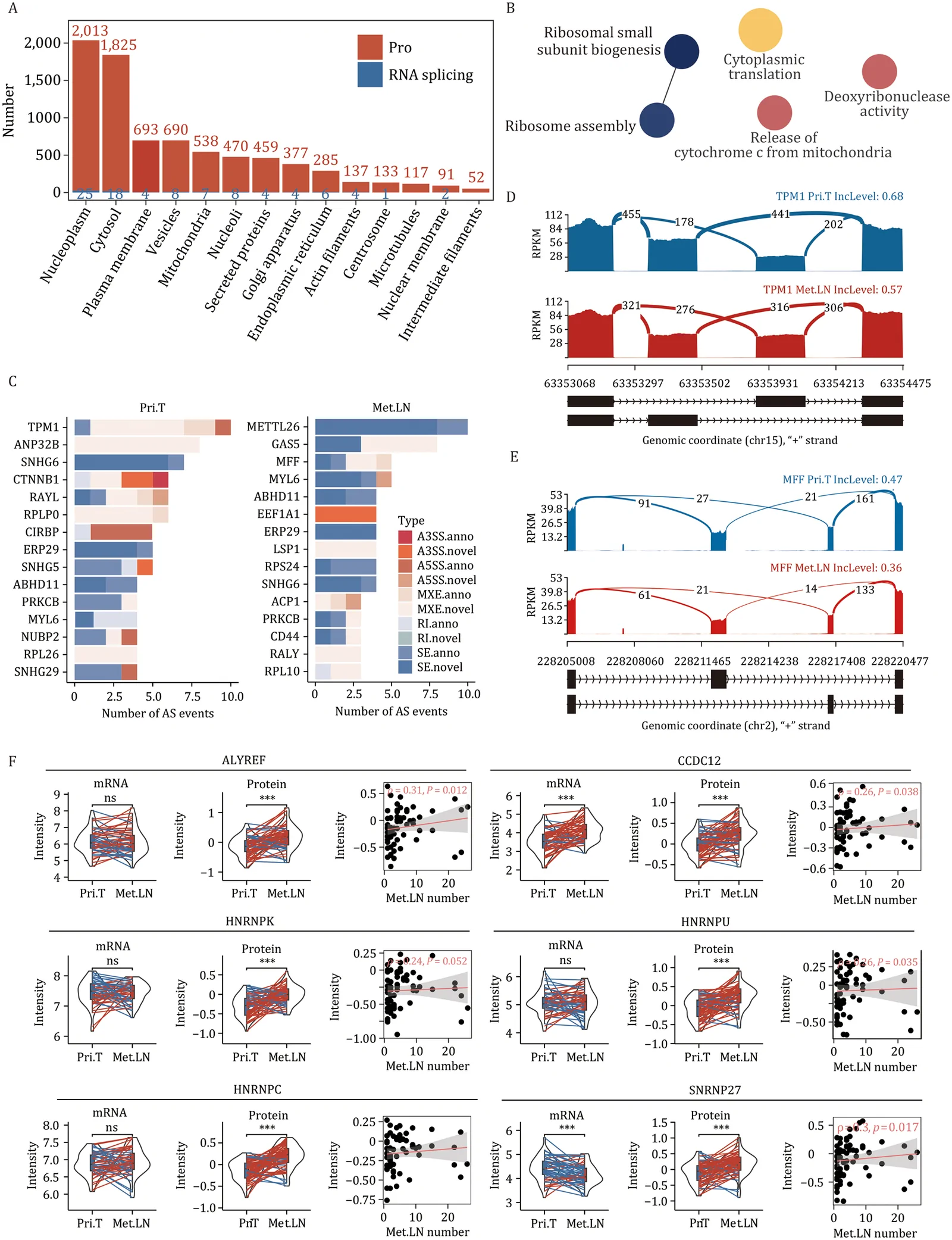
**Aberrant alternative splicing (AS) regulated early-stage breast cancer metastasis**. (A) Subcellular distribution of identified proteins (red) and proteins with AS at the gene expression level (blue), based on Gene Ontology. (B) GSEA-based KEGG pathway enrichment analysis of differentially expressed proteins involved in the spliceosome pathway between Pri.Ts (*n* = 65) and paired Met.LNs (*n* = 65). (C) High-frequency alterative splicing genes and in Pri.Ts (*n* = 55) and Met.LNs (*n* = 51). (D and E) Sashimi plots illustrating differentially quantified alternative splicing (AS) events occurring in *TPM1* (D) and *MFF* (E) between Pri.Ts and Met.LNs. (F) Comparison of transcriptional and protein expression levels of AS modulators, and their correlation with the number of lymph nodes metastasis, for differentially expressed proteins involved in the spliceosome pathway between Pri.Ts (*n* = 65 for proteome and *n* = 53 for transcriptome) and paired Met.LNs (*n* = 65 for proteome and *n* = 53 for transcriptome). ns represented *P* > 0.05, ***P < 0.001.

RNA-seq analysis identified a total of 742,279 splicing events, including exon skipping (SE), alternative 3′ splice sites (A3SS), alternative 5′ splice sites (A5SS), mutually exclusive exons (MEX), and retained introns (RI) (Table S3G). The proteins encoded by these alternatively spliced genes were distributed across various cellular organelles, with the highest proportion associated with actin filaments (Fig. 4A). KEGG pathway enrichment analysis indicated that these AS events were primarily involved in ribosome assembly and cytoplasmic protein translation (Fig. 4B). We analyze various types of AS events and conduct a statistical analysis to determine the association between different AS events and metastasis. Totally, 258 AS events exhibited significant differences between Pri.Ts and NATs (FDR < 0.1 and absolute IncLevelDifference > 0.1). Specifically, AS events were classified into two groups: (1) AS events significantly associated with metastasis, defined as those showing significant differences between Pri.Ts and NAT samples (FDR < 0.1 and absolute IncLevelDifference > 0.1) and significantly correlated with lymph node involvement in tumor samples (Spearman correlation, *P* < 0.05); and (2) Other AS events, which show significant differences between Pri.Ts and NAT samples (FDR < 0.1 and absolute IncLevelDifference > 0.1) but are not significantly correlated with lymph node involvement in tumor samples (Spearman correlation, *P* > 0.05). Among these, MXE was the most prevalent type (Fig. S4A). Additionally, the frequency and distribution of specific RNA AS events were comprehensively analyzed. Among genes exhibiting high-frequency AS events, *TPM1* and *ANP32B* showed significant differences in AS events in Pri.Ts, while *METTL26* exhibited the highest frequency of AS events in Met.LNs (Fig. 4C). Genes such as *RALY*, *MYL6*, and *PRKCB* demonstrated distinct AS event frequencies in both Pri.Ts and Met.LNs, and their corresponding mRNA and protein levels displayed significantly different expression patterns between Pri.Ts and Met.LNs (Fig. S4B–E). We collected splicing factors and RNA-binding regulatory proteins from the UniProt and GO databases and systematically examined their expression changes at both the transcriptomic and proteomic levels. Overall, the expression levels of these splicing-related regulatory factors were significantly higher in Met.LNs compared to Pri.Ts at the proteome level (Fig. S4F). In contrast, no clear or consistent differences were found at the transcriptome level. We identified that the *TPM1*, *MFF*, *SMTN*, *SEC31A*, and *DGUOK* genes exhibited significant differences. Notably, *TPM1*, *MFF*, and some genes including *SMTN* were frequently subjected to AS genes in both Pri.Ts or Met.LNs samples (Figs. 4D–E and S4G–H). In summary, we systematically analyzed the characteristics of splicing events in Met.LNs and Pri.Ts samples from breast cancer patients. We hypothesize that the AS modulates the expression of gene transcripts, potentially resulting in variations in protein expression levels.

The precision and diversity of AS events were influenced by several factors, including splice site strength, the concentration and combination of enhancing and silencing splicing factors, chromatin modifications, and RNA secondary structures (Jordan et al., 2019). We then analyzed the AS regulatory factors and found ALYREF, HNRNPK, HNRNPC, HNRNPU, CCDC12, and SNRNP27 were found to be significantly upregulated at the protein level in Met.LNs compared to Pri.Ts, without notable changes at the transcriptional level (Fig. 4F). Additionally, a significant positive correlation was observed between the abundance of most AS regulatory factors in Pri.Ts and the number of Met.LNs (Fig. 4F). These findings suggest that aberrant AS may serve as a key driver of breast cancer metastasis at the proteomic level, highlighting its potential as a therapeutic target for invasive breast cancer.

### Identification and validation of specific phosphosites as prognostic biomarkers

**Figure 5.**
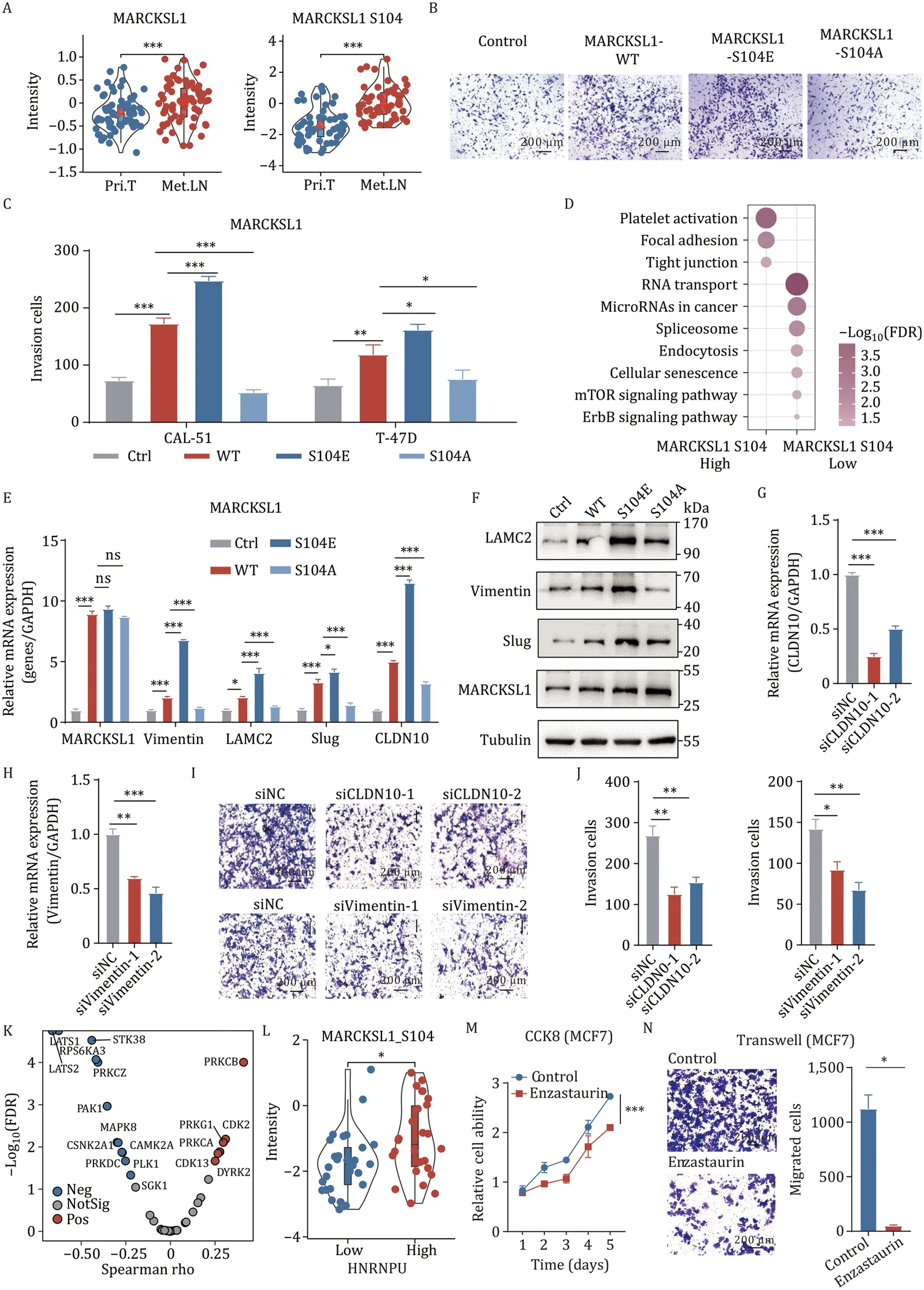
**Identification and validation of specific phosphosites as prognostic biomarkers**. (A) Comparison of MARCKSL1 protein and phosphorylation levels between Pri.T (*n* = 65 for proteome and *n* = 55 for phosphoproteome) and paired Met.LNs (*n* = 65 for proteome and *n* = 55 for phosphoproteome) (Wilcoxon signed-rank test). ***P < 0.001. (B) Cell migration experiments verify the impact of MARCKSL1 S104 phosphorylation levels on cancer cell migration. S104 is mutated to alanine (A) to mimic the phosphorylation-inhibited group, while S104 is mutated to glutamic acid (E) to mimic the phosphorylation-activated group. (C) Transwell migration assays assessing the effect of MARCKSL1-S104 phosphorylation on cell migration in CAL-51 and T-47D cells. Student’s *t*-test, *P < 0.05, **P < 0.01, ***P < 0.001. (D) KEGG pathway analysis comparing high MARCKSL1-S104 with low MARCKSL1 S104 groups using all Pri.T samples. (E) Real-time qPCR validation of representative *MARCKSL1* target genes in cells overexpressing *MARCKSL1*-WT versus S104E or S104A mutants. Student’s *t*-test, *P < 0.05, **P < 0.01, ***P < 0.001. (F) Western blot validation of *MARCKSL1* target genes in CAL-51 cells overexpressing *MARCKSL1*-WT compared to S104E or S104A mutants. (G) SiRNA transfection of *CLDN10* in MDA-MB-231 cells, with depletion efficiency validated by real-time qPCR. Data are presented as mean ± SEM (Student’s *t*-test), Student’s *t*-test, *P < 0.05, **P < 0.01, ***P < 0.001. (H) SiRNA transfection of Vimentin in MDA-MB-231 cells, with depletion efficiency validated by real-time qPCR. Data are presented as mean ± SEM (Student’s *t*-test), *P < 0.05, **P < 0.01, ***P < 0.001. (I) Impact of *CLDN10* and Vimentin knockdown on cancer cell migration. (J) Trans-well migration assays evaluating the effects of *CLDN10* and Vimentin expression on cell migration. Student’s *t*-test, *P < 0.05, **P < 0.01. (K) Correlation between kinase activity and phosphorylation level of MARCKSL1-S104 using all samples. Red circles denoted FDR < 0.05 and Spearman correlation coefficient > 0, and blue circles denoted FDR < 0.05 and Spearman correlation coefficient < 0. (L) Comparison of the phosphorylation level of MARCKSL1-S104 in samples with low HNRNPU expression versus those with high HNRNPU expression using all Pri.T samples. Wilcoxon rank-sum test, and *P < 0.05. (M) The CCK8 analysis was conducted to evaluate the effects of targeted inhibition of PRKCB activity on the migratory ability of MCF7 cancer cells. Student’s *t*-test, ***P < 0.001. (N) Cell migration assays confirm the effect of targeted PRKCB activity inhibition on the migratory behavior of MCF7 cancer cells. Student’s *t*-test, *P < 0.01.

Our proteomics and phosphoproteomics data analyses indicated that protein phosphorylation plays a critical regulatory role in breast cancer metastasis, and several potential marker modification sites were successfully identified. Based on this finding, we proceeded with mechanistic verification and functional characterization of these core phosphorylation sites through cell-based functional experiments and chemical intervention approaches. Focusing specifically on phosphorylation events, FKBP15 and MARCKSL1 were identified as potential key regulators of lymph node metastasis according to functional prediction scores. Mass spectrometry revealed significantly higher phosphorylation levels of MARCKSL1-S104 and FKBP15-S320 in Met.LNs compared to Pri.Ts, while the total protein expression levels of these proteins did not differ significantly (Figs. 5A and S5A). MARCKSL1 is known to influence cell migration and invasion by interacting with F-actin and cortactin, thereby affecting invadopodia formation and extracellular matrix (ECM) degradation (Zhao et al., 2023); it also contributes to resistance to docetaxel (Jiang et al., 2022). FKBP15, a member of the FKBP-type peptidyl-prolyl cis-trans isomerase (PPIase) family, is involved in actin filament organization and cell spreading (Harbour et al., 2012; Luan et al., 1996; Pan et al., 2010; Viklund et al., 2008, 2009), although its role in cancer-related post-translational modifications is less understood. To validate the observed elevation in phosphorylation modification levels of MARCKSL1 at Ser104 and FKBP15 at Ser320 in Met.LNs relative to Pri.Ts samples, we performed quantitative analysis using the parallel reaction monitoring (PRM) technique. We synthesized phosphorylated peptide standards for two sites (LSGLS[phos]FK for MARCKSL1-S104 and DSAAPSPIPGADNLS[phos]ADPVVSPPTSIPFK for FKBP15-S320). Using PRM-based quantitative proteomics, we analyzed 30 paired tumor and metastatic lymph node samples to compare phosphorylation levels (Fig. S5B). Both MARCKSL1 S104 and FKBP15 S320 phosphorylation were significantly higher in Met.LNs than in Pri.Ts. Phosphorylation of MARCKSL1-S104 increased in 57% (17/29) of Met.LNs (ratio > 1.5), and FKBP15-S320 phosphorylation rose in 67% (20/30). These results confirmed ­elevated phosphorylation at MARCKSL1-S104 and FKBP15-S320 in Met.LNs, consistent with our proteomic data.

To validate the roles of MARCKSL1-S104 and FKBP15-S320 phosphorylation in breast cancer metastasis, we first assessed the endogenous levels of MARCKSL1 and FKBP15 in breast cancer cell lines. Subsequently, wild-type (WT), mimic phosphorylation-activated (S104E, S320E), and phosphorylation-inhibited mutants (S104A, S320A) were overexpressed in MDA-MB-231 cells (Figs. 5B, 5C, S5C, and S5D). The S104E mutant of MARCKSL1 significantly increased cell migration compared to the WT, while the S104A mutant decreased migration. Similarly, phosphorylation of FKBP15 at S320 enhanced cancer cell migration (Fig. S5E). These findings suggest that the phosphorylation levels of MARCKSL1-S104 and FKBP15-S320 may play the important roles in the progression and metastasis of breast cancer to lymph nodes.

To explore the potential molecular mechanism, samples were stratified into high and low MARCKSL1-S104 phosphorylation groups according to the phosphorylation level of MARCKSL1-S104. KEGG pathway analysis indicated that MARCKSL1-S104 phosphorylation upregulated pathways associated with cell migration, including platelet activation signaling, tight junction, and focal adhesion pathways (Fig. 5D). Similarly, FKBP15-S320 phosphorylation influenced the Apelin signaling and tight junction pathways (Fig. S5F). Four genes known to promote cancer cell migration—CLDN10, Vimentin, Slug, and LAMC2—were selected for further analysis. RT-qPCR and western blotting demonstrated that high phosphorylation of MARCKSL1-S104 or FKBP15-S320 significantly increased the mRNA and protein levels of these genes (Figs. 5E, 5F, and S5G–H). Additionally, knockdown of CLDN10 and Vimentin reduced cell migration in MARCKSL1-overexpressing MDA-MB-231 cells (Fig. 5G–J).

To find the potential up-stream kinase of these phosphosites, we applied the ssGSEA approach to estimate kinase activity using kinase-substrates data from PhosphoSitePlus database. Further analysis revealed a positive correlation between the phosphorylation levels of MARCKSL1-S104 and FKBP15-S320 and the activity of kinases such as PRKCA, PRKCB, CDK2, and CDK13 (Figs. 5K and S5I). Notably, a significant difference in PRKCB expression was observed between Pri.Ts with high and low metastatic lymph node counts (Fig. S5J), suggesting that MARCKSL1-S104 and FKBP15-S320 could be potential substrates. We then used synthetic peptides containing the target sites and performed *in vitro* incubation with recombinant PRKCB and then analyzed by mass spectrometry. Mass spectrometry revealed an 80 Da mass difference between the unmodified and in vitro incubation-generated peptide, consistent with phosphorylation (Fig. S6A and S6B). Phosphorylation-induced changes in hydrophilicity resulted in distinct retention times. MS/MS analysis enabled clear identification of phosphorylated peptides and precise localization of modification sites (Fig. S6C–D). To validate the *in vitro* kinase assay results, we synthesized phosphorylated peptide standards (LSGLS[phos]FK and DSAAPSPIPGADNLS[phos]ADPVVSPPTSIPFK) for comparison. MS/MS spectra of assay-derived and synthetic peptides showed highly similar fragment ion patterns, confirming structural integrity and accurate phosphorylation. Notably, although the unmodified peptides in this study contained two or more serine (S) residues, phosphorylation occurred only at the intended sites, highlighting PRKCB’s high catalytic specificity.

Additionally, 12 AS regulatory factors were identified as correlating with PRKCB activity, with HNRNPU ranking highest (Fig. S6E). PRKCB activity was also elevated in Pri.Ts with high HNRNPU abundance, indicating that AS events may influence PRKCB activity. Additionally, the phosphorylation levels of MARCKSL1-S104 and FKBP15-S320 were significantly higher in samples with elevated HNRNPU expression (Figs. 5L and S6F). We then knockdown the *HNRNPU* gene using small interfering RNA (siRNA) and successfully established *HNRNPU* knockdown MCF7 cell lines (Fig. S6G). Cell migration assays demonstrated that *HNRNPU* silencing significantly inhibited the migratory capacity of MCF7 cells (Fig. S6H). To further elucidate the molecular mechanism, we employed PRM technology to quantify the phosphorylation levels of MARCKSL1-Ser104 and FKBP15-Ser320 in *HNRNPU* knockdown cells and their corresponding control cells. The results revealed that phosphorylation of both MARCKSL1-S104 and FKBP15-S320 was significantly reduced in HNRNPU-silenced cells compared to controls (Fig. S6I, Student’s *t*-test). Collectively, these findings—from cell migration assays and PRM-based phosphorylation quantification—demonstrated that the splicing factor HNRNPU regulates the phosphorylation dynamics of MARCKSL1-S104 and FKBP15-S320, and this regulatory role is functionally linked to the migratory capacity of breast cancer cells.

To verify whether PRKCB plays a key role in the lymph node metastasis of breast cancer, we conducted cell-level intervention experiments using Enzastaurin, a selective inhibitor of PRKCB. The results demonstrated that the addition of the PRKCB selectively inhibitor significantly suppressed the proliferation and migration of MCF7 and MB-231 breast cancer cells (Figs. 5M, 5N, S6J, and S6K).

Overall, these findings suggest that MARCKSL1-S104 and FKBP15-S320 phosphorylation were mediated by PRKCB and might influenced by AS events, represent potential mechanism and biomarkers for lymph node metastasis.

### Immune characteristics of primary tumors and paired metastatic lymph nodes

Immunity plays a crucial role in tumor development and metastasis. Our study provides a comprehensive omics dataset encompassing multiple molecular dimensions of breast cancer lymph node metastasis, facilitating in-depth investigation into the molecular characteristics of breast cancer lymphatic metastasis. Furthermore, we conducted a systematic analysis of the immune features associated with the breast cancer metastasis process.

**Figure 6.**
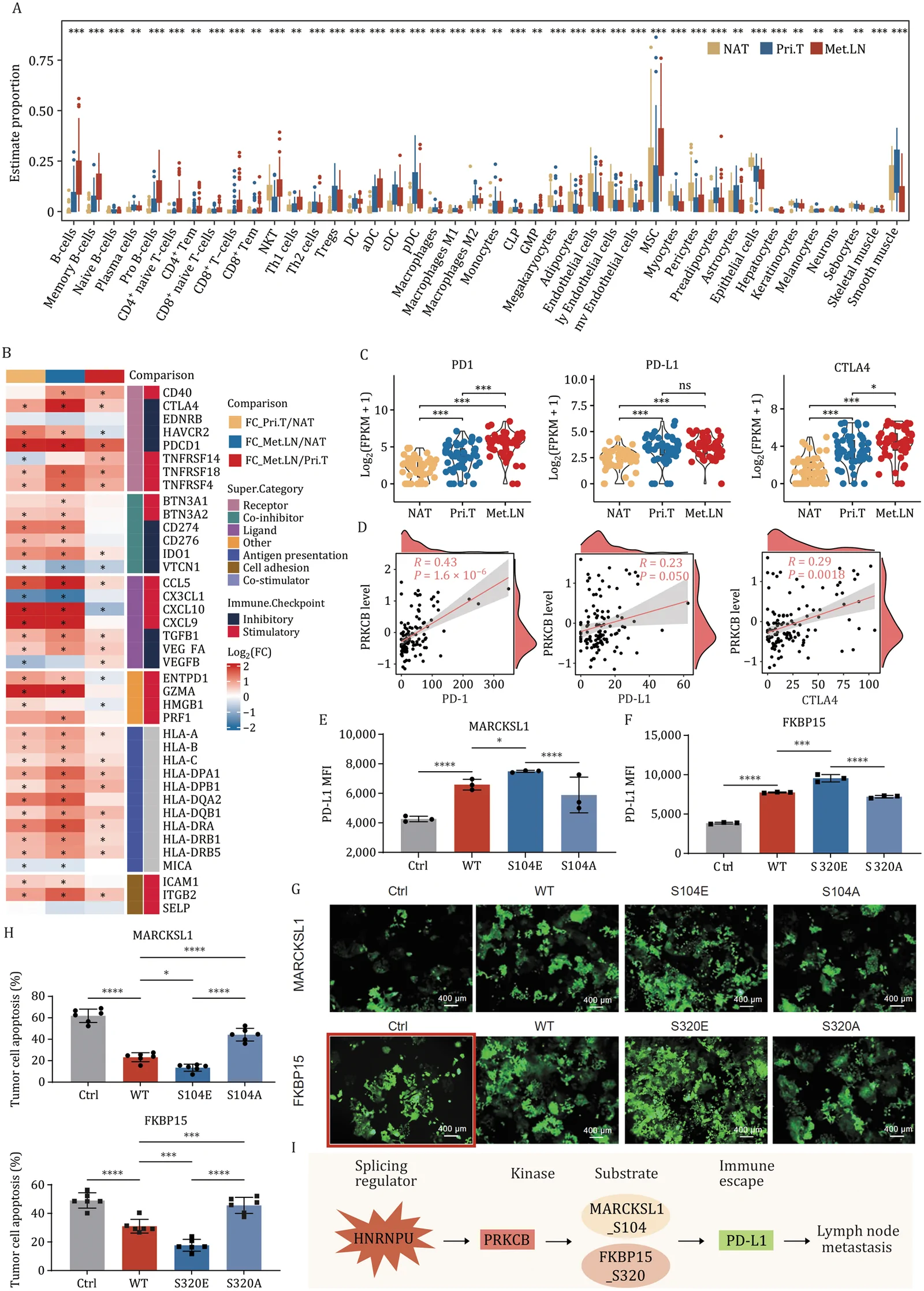
**Immune characteristics of primary tumors and paired metastatic lymph nodes**. (A) Comparison of xCell-derived immune cell signatures between normal adjacent tissues (NATs) (*n* = 51), primary tumors (Pri.Ts) (*n* = 55) and Met.LNs (*n* = 58) using all samples (*P < 0.05, **P < 0.01, adjusted *P*-value from Wilcoxon rank sum and signed rank test). Tem, effector memory T cells; Tcm, central memory T cells; DC, dendritic cell. (B) Heatmap illustrating differentially expressed proteins between NATs (*n* = 51), Pri.Ts (*n* = 55), and Met.LNs (*n* = 58) using all samples involved in MHC molecules, stimulatory and inhibitory immune modulators (Wilcoxon signed-rank test). *P < 0.05. (C) Comparison of PD-1, PD-L1, and CTLA4 expression between NATs (*n* = 50), Pri.Ts (*n* = 50), and Met.LNs (*n* = 50) using all samples (Wilcoxon signed-rank test). *P < 0.05. **P < 0.01. ***P < 0.001. ns represented *P* > 0.05. (D) Scatterplot showing Spearman’s correlation of PD-1, PD-L1, and CTLA4 expression and PRKCB levels using all samples. (E and F) Mean fluorescence intensity (MFI) in *MARCKSL1* wild-type and mutated cells (E), as well as in *FKBP15* wild-type and mutated cells (F), is presented as mean ± SD. A one-way ANOVA test was used for statistical analysis. *n* = 3 for each group. (G) Representative images of *MARCKSL1* wild-type and mutant cells (top), as well as *FKBP15* wild-type and mutant cells (bottom), expressing GFP fluorescence in co-culture systems. (H) The extent of cell apoptosis in *MARCKSL1* wild-type and mutant cells (F), as well as in *FKBP15* wild-type and mutant cells (G), was assessed by lactate dehydrogenase (LDH) assay following co-culture with cytotoxic T cells. Apoptosis levels were quantified based on LDH release, and *P*-values were determined using a two-sided unpaired Student’s *t*-test (*n* = 6 independent cell culture replicates). Data are presented as mean ± standard deviation (SD). (I) A scheme showed the potential regulation relationship proposed by the manuscript.

We used xCell to evaluate the levels of immune cell infiltration (Becht et al., 2016), and the results demonstrated differential immune cell infiltration across NATs, Pri.Ts, and Met.LNs (Fig. 6A). Compared to Pri.Ts, Met.LNs exhibited stronger signatures of both cytotoxic immune cells, including CD8^+^ T cells, B cells, natural killer cells, activated dendritic cells (aDCs), and Th1 cells, as well as immune inhibitory cells such as regulatory T cells and M2 macrophages (Fig. 6A). This is consistent with the pathway enrichment results of differentially expressed proteins shown in Fig. 2D, which indicates that Met.LNs showed upregulation of multiple immune-related pathways, including the JAK-STAT signaling pathway, B cell receptor signaling pathway, and natural killer cell-mediated cytotoxicity. Furthermore, xCell-derived cell type enrichment scores were used to assess the infiltration of major immune cells, such as CD8^+^ T cells and dendritic cells (DCs), during the cancer-immunity cycle in different immunohistochemistry (IHC) subtypes. Subtype-specific analysis revealed that anti-cancer immune cells, such as activated DCs, classical DCs, and cytotoxic CD8^+^ T cells, were more or less upregulated in Met.LNs, mainly in Luminal and HER2-positive subtypes (Figs. S7A and S7B). However, no significant differences in immune cell infiltration were detected between Met.LNs and Pri.Ts in triple-negative breast cancer (TNBC) subtype. We speculate that the tumor microenvironment of Luminal breast cancer is characterized by a lower infiltration of immune cells, presenting an immune-cold state; while the *in situ* tumor of TNBC tumors showed significant immune cell infiltration due to a higher mutational burden, presenting an immune-hot state.

Notably, MHC molecules involved in antigen presentation, along with stimulatory and inhibitory immune modulator proteins, including receptor and ligand expression, were upregulated in primary Pri.Ts compared to NATs, with these differences being even more pronounced in Met.LNs (Fig. 6B). Additionally, these significant differences between Met.LNs and Pri.Ts were predominantly observed in the Luminal subtype (Fig. S7C). Given that neither cancer antigen presentation nor T cell infiltration appeared impaired, a potential obstacle may lie in the process of cancer cell elimination. We also found that immune checkpoint molecules, such as CTLA4, PD-L1, PD-1, and IDO1, were upregulated in Pri.Ts compared to NATs, and this upregulation was maintained or enhanced in Met.LNs relying on a subtype-specific manner (Figs. 6C and S7D–F). This result aligns with the PTM-SEA analysis of differentially phosphorylated modification sites presented in Fig. S3E, suggesting that immunosuppressant drugs, such as sirolimus, exert significant and specific perturbation effects within the luminal subtype. Therefore, we speculate that immunotherapies may benefit breast cancer patients with lymph node metastasis, particularly those with the Luminal subtype.

To further investigate potential determinants of immune escape during lymph node metastasis, correlation analysis was performed using transcriptomic and proteomic data. As expected, the expression of PD-1, PD-L1, and CTLA4 was positively correlated with the levels of PRKCB, and HNRNPU, respectively (Figs. 6D and S7F). These findings suggest that a high level of PRKCB expression, which catalyzes the phosphorylation of FKBP15 at Ser320 and MARCKSL1 at Ser104, may contribute to immune suppression through the upregulation of immune checkpoint expression in metastatic lymph nodes. To further validate the hypothesize that the effects of MARCKSL1-S104E and FKBP15-S320 phosphorylation on the expression of immune checkpoint molecules such as PD-L1, as well as their impact on tumor cell apoptosis, flow cytometry was first employed to evaluate PD-L1 expression levels in cells expressing wild-type MARCKSL1 versus the S104 mutant, and in cells expressing wild-type FKBP15 versus the S320 mutant. The results demonstrated that under conditions mimicking phosphorylation modification, PD-L1 expression was significantly upregulated in MARCKSL1-S104E mutant cells compared to the wild-type control. Similarly, a significant increase in PD-L1 expression was observed in FKBP15-S320E mutant cells relative to their wild-type counterpart (Figs. 6E, 6F, S7H, and S7I). To further investigate the functional impact of these phosphorylation events on tumor immune escape, we conducted co-culture experiments using CD8^+^ T cells and the aforementioned MARCKSL1/FKBP15 WT or mutant cell lines. These experiments revealed that under conditions mimicking phosphorylation (S→E mutation), tumor cell apoptosis was significantly decreased; in contrast, under conditions simulating reduced phosphorylation (S→A mutation), tumor cell apoptosis was markedly increased (Fig. 6G–H).

Collectively, these findings and proteogenomic data indicate that the upregulation of PRKCB may increase the phosphorylation levels of MARCKSL1 at Ser104 and FKBP15 at Ser320, thereby modulating the expression of immune checkpoint molecules such as PD-L1, enhancing immune suppression, and protecting tumor cells from immune cell-mediated cytotoxicity (Fig. 6I). These results underscore the potential regulatory role of these phosphorylation events in shaping the tumor immune microenvironment, suggesting that patients with breast cancer exhibiting lymph node metastasis—particularly those with the Luminal subtype—may benefit therapeutically from immunotherapy.

## Discussion

A critical issue in breast cancer research is the direct understanding of the molecular mechanisms underlying metastasis, which is essential for advancing precision medicine (Chen et al., 2023b). Traditional research methods have primarily investigated breast cancer by focusing on specific proteins or pathways (Elango et al., 2024; Guo et al., 2024; Li et al., 2024), often providing only single-dimensional views at the genetic or translational level, and overlooking the functional importance of proteins (Kadamkulam Syriac et al., 2024). Thus, there is an urgent need to collect paired metastatic samples from breast cancer patients to offer a more comprehensive perspective and accelerate both clinical and basic research.

In this study, multidimensional data from Pri.Ts and paired Met.LNs were integrated to create a comprehensive multi-omics resource, encompassing genomic, transcriptomic, proteomic, and phosphoproteomic data. This approach elucidated the molecular features and mechanisms of breast cancer lymph node metastasis. Genomic and transcriptomic analyses have expanded our understanding of the molecular characteristics of this aggressive process (Jovanovic et al., 2024; Nguyen et al., 2019). Through the systematic integration and in-depth analysis of multi-omics data, we observed that the regulatory impact of gene-level mutations on the expression of downstream transcriptome and proteome is relatively limited. To a certain extent, transcriptomic and proteomic data exhibit a high degree of consistency, collectively identifying the key pathways that undergo significant alterations during breast cancer lymphatic metastasis. However, proteomic and post-translational modification (PTM) profiling revealed more distinct and functionally significant variations, which have a direct impact on biological functions (Vu et al., 2018; Zhai et al., 2022). Our analysis of multi-dimensional omics data, combined with previous studies, demonstrated that specific alterations in protein expression are highly associated with gene amplification, deletion, and transcriptional regulation (Chick et al., 2016; Hu et al., 2023). Interestingly, many proteins that underwent splicing at gene level did not show noticeable variations at the genetic or transcriptional levels. This suggests that transcriptional dynamics may not fully translate to protein or phosphorylation levels (Schwanhäusser et al., 2011). We hypothesize that additional mechanisms, beyond genomic and transcriptomic alterations, may contribute to the intrinsic molecular characteristics of breast cancer metastasis.

Through integrated global differential analysis of transcriptomics, proteomics, and phosphoproteomics data, along with pathway-level characterization, our study revealed that AS-related pathways were significantly enriched at both the protein and phosphorylation levels, a phenomenon that was not evident at the transcriptional level (Wu et al., 2022; Zhao et al., 2022). Phosphorylation plays a critical role in the regulation of intracellular signal transduction pathways. Based on large-scale paired tumor tissue and lymphoid tissue samples collected in this study, we systematically explored potential biomarkers associated with phosphorylation modification sites. Finally, 62 key phosphorylation sites associated with metastasis were identified, predominantly involved in AS pathways. In Met.LNs, phosphorylation levels of MARCKSL1-S104 and FKBP15-S320 were significantly elevated. MARCKSL1 has been shown to influence cell migration and invasion by interacting with F-actin and cortactin, thereby affecting extracellular matrix degradation and resistance to docetaxel (Jiang et al., 2022; Zhao et al., 2023). FKBP15, a member of the FKBP-type PPIase family, plays a role in actin filament organization and cell spreading, although its involvement in cancer-related post-translational modifications remains poorly understood (Fan et al., 2018). We further carried out cellular-level experiments to explore whether changes in the phosphorylation status of key residues could influence cell proliferation, and to verify whether tumor metastasis could be suppressed through pharmacological modulation of its upstream kinases. As a result, cell-based assays confirmed that the phosphorylation levels of MARCKSL1 at serine 104 (S104) and FKBP15 at serine 320 (S320) play a crucial role in regulating cell proliferation and migration. Moreover, inhibition of the upstream kinase PRKCB was found to significantly attenuate the proliferative and migratory capacity of breast cancer cells.

Immunity plays a critical role in cancer development, and clinical data suggest that predictive biomarkers and combination therapies can contribute to improved outcomes (Adams et al., 2019; Huang et al., 2024). Proteogenomic analysis enabled us to characterize the immune landscape of lymph node metastasis in breast cancer (Johansson et al., 2019). Notably, patients exhibited stronger immune signals in their lymph node metastasis compared to their primary tumors, while tumor cells in Met.LNs promoted the upregulation of immune checkpoint molecules such as PD-1, PD-L1, CTLA4, and IDO1 to contribute to immune suppression, offering novel insights for future immunotherapies.

Despite its contributions, this study has several limitations. The limited sample size for multidimensional omics analysis hindered the ability to distinguish molecular characteristics and diagnostic potential across different IHC subtypes. Additionally, the relatively short follow-up period for clinical prognosis data constrained the scope of metastasis prognosis analysis. Although correlations between molecular dimensions were identified, direct biological validation is still required. Nonetheless, the multi-omics approach has enriched the current understanding of breast cancer metastasis and complemented existing research.

In conclusion, this study establishes a comprehensive multi-omics data repository for breast cancer accompanied by lymph node metastasis, offering a systematic characterization of the molecular landscapes and distinctive features of primary breast tumors and their corresponding metastatic lymph nodes. Through an integrative multi-omics analysis, the study revealed the unique proteome and phosphoproteome features of breast cancer metastasis to lymph nodes. We also found that regional lymph node metastasis is predominantly associated with alterations in the proteome and phosphoproteome, rather than by genomic or transcriptomic changes. We identified critical phosphorylated sites associated with breast cancer lymph node metastasis, including MARCKSL1-S104 and FKBP15-S320. These modifications are regulated by members of the PKC kinase family. Inhibiting PRKCB activity effectively suppresses breast cancer cell proliferation and migration. Immune profiling also showed correlations between phosphorylated proteins and immune checkpoints, suggesting immune evasion in metastasis. Overall, this study provides a valuable multidimensional molecular data resource of breast cancer metastasis to lymph nodes, which contributes to a more comprehensive molecular understanding of the disease and provides potential advancements in diagnostics and precision therapeutic approaches for breast cancer lymph node metastases.

## Supplementary Material

pwag002_Supplementary_Data

## Data Availability

The mass spectrometry raw files and the database search result files related to proteomics and phosphoproteomics have been deposited to the ProteomeXchange Consortium via the iProX partner with the project ID IPX0009341001. The raw sequence data reported in this paper have been deposited in the Genome Sequence Archive (Genomics, Proteomics & Bioinformatics 2021) in National Genomics Data Center (Nucleic Acids Res 2022), China National Center for Bioinformation/Beijing Institute of Genomics, Chinese Academy of Sciences (GSA-Human: HRA008454) that are publicly accessible at ngdc.cncb.ac.cn/gsa-human.

## Code availability

No novel source code was presented in this study.

## Resource availability

### Lead contact

Further information and requests for resources and reagents should be directed to and will be fulfilled by the lead contact, K.-D. Yu (yukeda@fudan.edu.cn).

### Materials availability

Further information and requests for resources and reagents should be directed to and will be fulfilled by the Lead Contact, K.-D. Yu (yukeda@fudan.edu.cn) with a completed Materials Transfer Agreement. This study did not generate new unique reagents.
