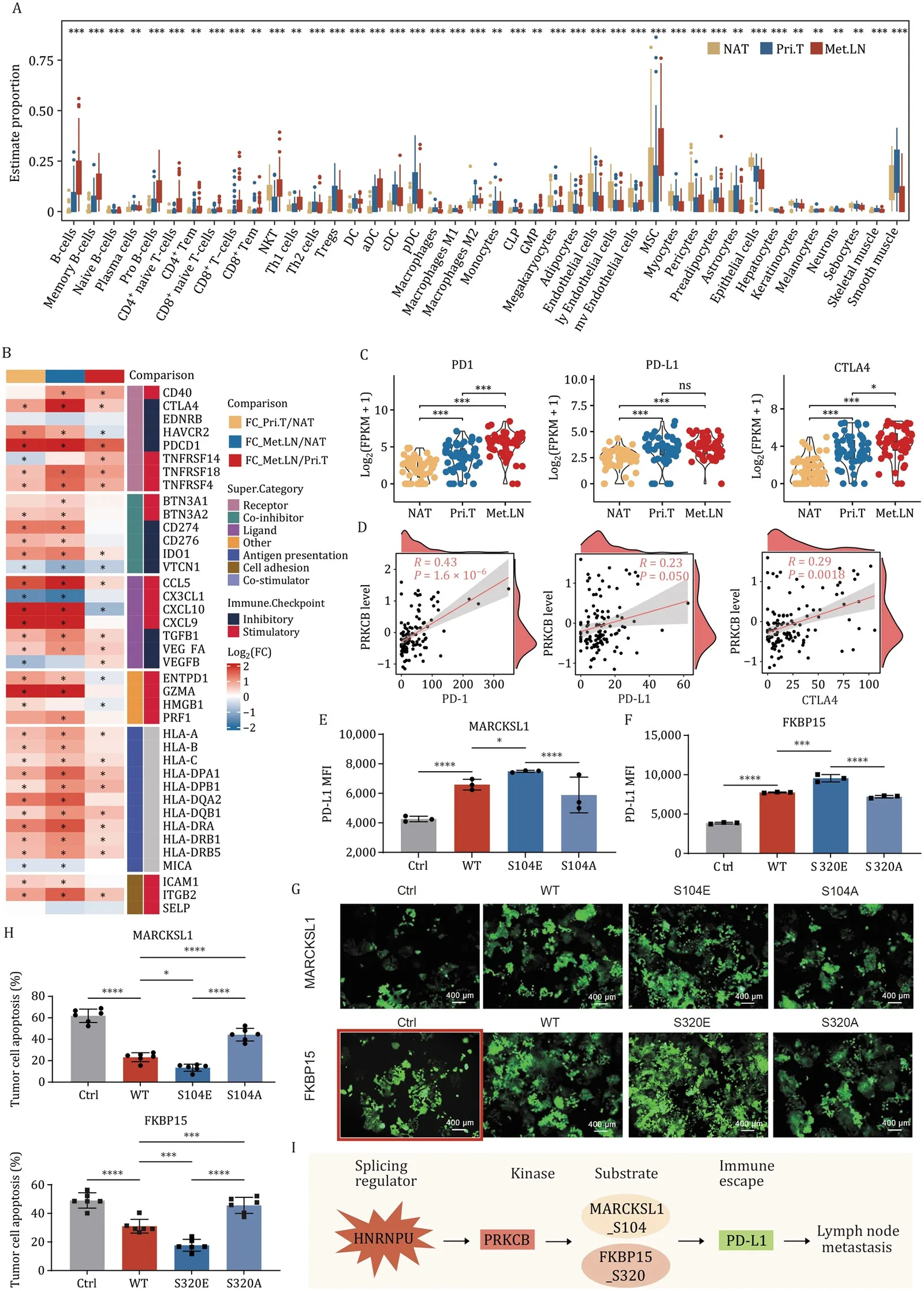# Correction to: Multi‐omics analysis reveals comprehensive aberrant protein and phosphorylation characteristics in breast cancer and paired metastatic lymph nodes

**DOI:** 10.1093/procel/pwag055

**Published:** 2026-08-28

This is a **correction** to:

Linhui Zhai, Cui-Cui Liu, Lei Zhao, Le-Wei Zheng, Chengyu Chu, Hong Hu, Yu-Wen Cai, Lie Chen, Yi-Ming Liu, Yiou Wang, Wensi Zhao, Yuqi Huang, Shiyu Duan, Zhi-Ming Shao, Yiting Jin, Minjia Tan, Ke-Da Yu, Multi‐omics analysis reveals comprehensive aberrant protein and phosphorylation characteristics in breast cancer and paired metastatic lymph nodes, *Protein & Cell*, 2026; pwag002, https://doi.org/10.1093/procel/pwag002

In the originally published version of this manuscript, authors identified an inadvertent duplication of cell images between the MARCKSL1 WT group and the FKBP15 Ctrl group in Fig. 6G. The cell image for the FKBP15 Ctrl group has been updated, with the corrected area marked by a red box. This correction does not impact the study’s conclusions or discussion.

**Figure:**