# Supplementary material for: Multi‐omics analysis reveals comprehensive aberrant protein and phosphorylation characteristics in breast cancer and paired metastatic lymph nodes
Source: Protein Cell. 2026 Jan 12;17(9):821–39. doi: 10.1093/procel/pwag002 (PMC13521278; doi:10.1093/procel/pwag002)
Supplement: Supplementary file 1 [file pwag002_Supplementary_Data.zip › PAC-25497-YKD-Supplementary File.pdf]

Supplemental information

Proteogenomics Reveal Comprehensive Aberrant Protein and Phosphorylation Characteristics in Breast Cancer and Paired Metastatic Lymph Nodes

Linhui Zhai[1, 2, 3, #], Cui-Cui Liu[1, #], Lei Zhao[2, 4, 5, #], Le-Wei Zheng[1, 6, #], Chengyu Chu[4,#] , Hong Hu[7,#], Yu-Wen Cai[1], Lie Chen[1], Yi-Ming Liu[1], Yiou Wang[1], Wensi Zhao[7], Yuqi Huang[4], Shiyu Duan[3], Zhi-Ming Shao[1], Yiting Jin[4, *], Minjia Tan[3, 4, 5, *], Ke-Da Yu[1, 6, *]

1. Department of Breast Surgery, Key Laboratory of Breast Cancer in Shanghai, Fudan University Shanghai Cancer Center and Cancer Institute, Department of Oncology, Shanghai Medical College, Fudan University, Shanghai 200032, China;
2. Translational Research Institute of Brain and Brain-Like Intelligence, Shanghai Fourth People's Hospital, and Cancer Center, School of Medicine, Tongji University, Shanghai 200434, China;
3. State Key Laboratory of Drug Research, Shanghai Institute of Materia Medica, Chinese Academy of Sciences, Shanghai 201203, China;
4. Department of Thyroid and Breast Surgery, General Surgery, Huashan Hospital, Fudan University, Shanghai 200040, China;
5. Zhongshan Institute for Drug Discovery, Shanghai Institute of Materia Medica, Chinese Academy of Sciences, Zhongshan, Guangdong 528400, China;
6. Shanghai Institute of Infectious Disease and Biosecurity, Fudan University, Shanghai 200040, China;
7. Division of Breast Surgery, Department of General Surgery, Shenzhen People's Hospital (The Second Clinical Medical College, Jinan University; The First Affiliated Hospital,Southern University of Science and Technology), Guangdong 518020, China;
8. Department of Thoracic Surgery, Shanghai Pulmonary Hospital, School of Medicine, Tongji University, Shanghai, 200433, China.

* Correspondence to:

Ke-Da Yu, Department of Breast Surgery, Shanghai Cancer Center and Cancer Institute, Department of Oncology, Shanghai Medical College, Fudan University, Shanghai 200032, China; E-mail: yukeda@fudan.edu.cn;
Minjia Tan, State Key Laboratory of Drug Research, Shanghai Institute of Materia Medica, Chinese Academy of Sciences, Shanghai 201203, China; E-mail: mjtan@simm.ac.cn;
Yiting Jin, Department of Thyroid and Breast Surgery, General Surgery, Huashan Hospital, Fudan University, Shanghai 200040, China; E-mail: jinyiting@huashan.org.cn.

Figure. S1.

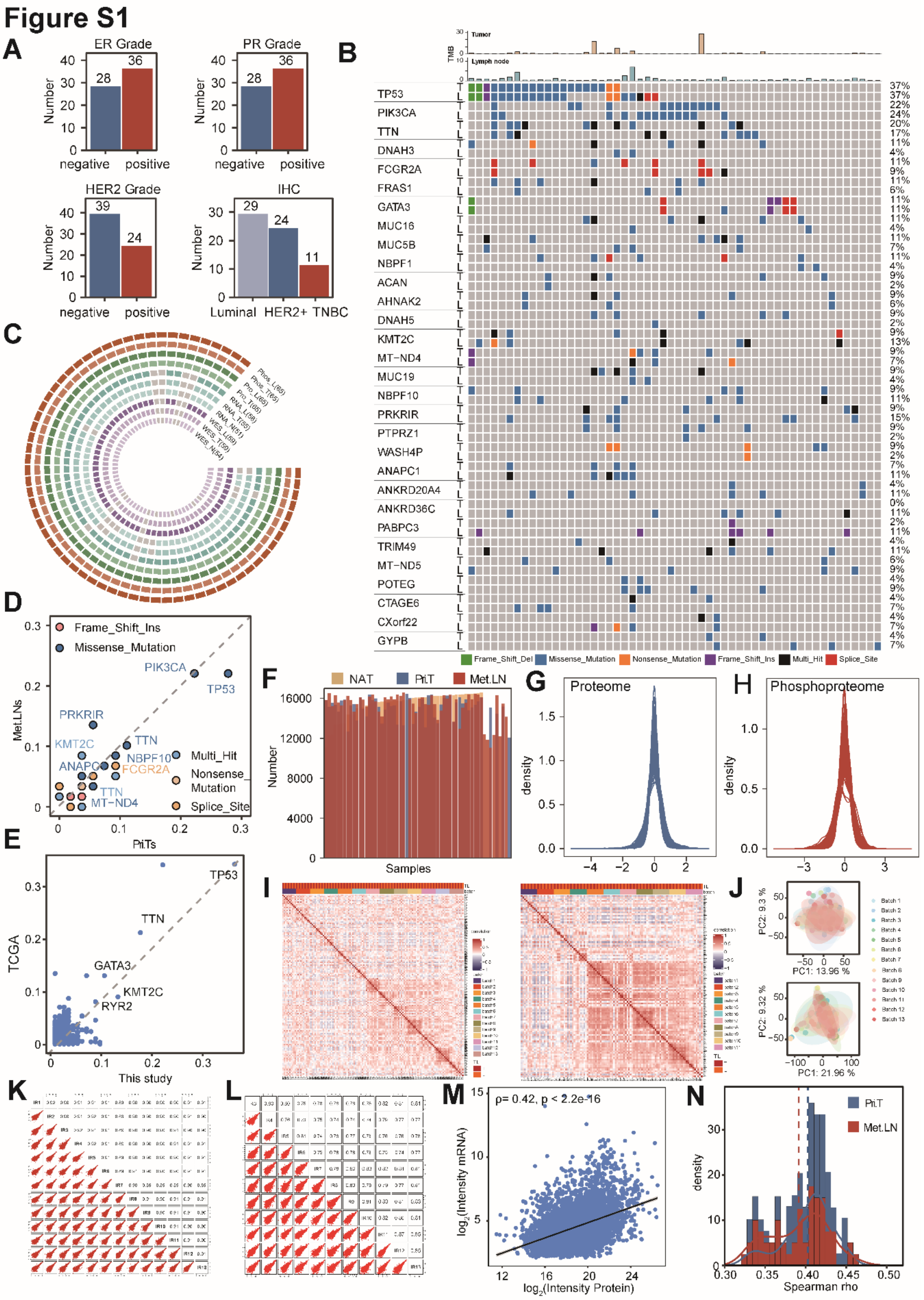
Figure S1
A
ER Grade
PR Grade
HER2 Grade
IHC
Number
negative
positive
Luminal
HER2+
TNBC
28
36
39
24
29
11
B
TMB
Tumor
Lymph node
TP53
PIK3CA
TTN
DNAH3
FCGR2A
FRAS1
GATA3
MUC16
MUC5B
NBPF1
ACAN
AHNAK2
DNAH5
KMT2C
MT−ND4
MUC19
NBPF10
PRKRIR
PTPRZ1
WASH4P
ANAPC1
ANKRD20A4
ANKRD36C
PABPC3
TRIM49
MT−ND5
POTEG
CTAGE6
CXorf22
GYPB
Frame_Shift_Del
Missense_Mutation
Nonsense_Mutation
Frame_Shift_Ins
Multi_Hit
Splice_Site
C
D
Frame_Shift_Ins
Missense_Mutation
Multi_Hit
Nonsense_Mutation
Splice_Site
Met.LNs
PriTs
E
TCGA
This study
TP53
TTN
GATA3
KMT2C
RYR2
F
NAT
Pri.T
Met.LN
Number
Samples
G
Proteome
density
H
Phosphoproteome
I
J
PC1: 13.96 %
PC2: 9.3 %
PC1: 21.96 %
PC2: 9.32 %
K
L
M
ρ= 0.42, p < 2.2e-16
log2(Intensity mRNA)
log2(Intensity Protein)
N
Pri.T
Met.LN
density
Spearman rho

Figure S1. Comprehensive Analysis of Proteogenomics Data in Primary Tumors and Paired Metastatic Lymph Nodes, Related to Figure 1.

A. Annotation of ER, PR, HER2 status, and Statistical comparison of different IHC grades for Pri.Ts and Met.LNs.

B. Number of tissue samples used for genomics, transcriptomics, proteomics, and phosphoproteomics analyses, reflecting those that passed quality control (QC) metrics.

C. The genomic profiles. Top: mutation counts of mutated genes in each sample. Bottom: the mutated genes and their occurrence in all Pri.Ts and Met.LNs tumor samples. Mutation percentages are indicated on the right.

D, E. Comparative analysis of mutation frequencies of highly mutated genes between Pri.Ts (n = 54) and Met.LNs (n = 59) (D) and a comparison of our dataset with TCGA mutation data (E).

F. Number of identified transcripts in NATs (n = 51, yellow), Pri.Ts (n = 55, blue), and Met.LNs (n = 58, red).

G, H. Data distribution for proteome (n = 130) (G) and phosphoproteome (n = 130) (H) across all tissue samples.

I. Heatmap illustrating batch effects among different TMT-labeling groups in proteome (n = 130, left) and phosphoproteome (n = 130, right) profiling.

J. PCA analysis results show batch effects removing among the proteome (top) and phosphoproteome (bottom) after batch effect correction.

K, L. Correlation analysis of 12 internal standard samples used in 12-batch TMT-labeling in proteomics data (K) and phosphoproteomics data (L), assessing the robustness of TMT-based quantification. Spearman’s correlation coefficients are shown in the top-right quadrant, with pairwise comparisons visualized in scatterplots in the bottom-left quadrant.

M, N. Scatter plot of the Spearman correlation between gene transcriptome expression and protein expression (M) and the comparison of Met.LNs (n = 58) and Pri.Ts (n = 55) sample-wise correlation between mRNA and protein expression (N).

Figure. S2.

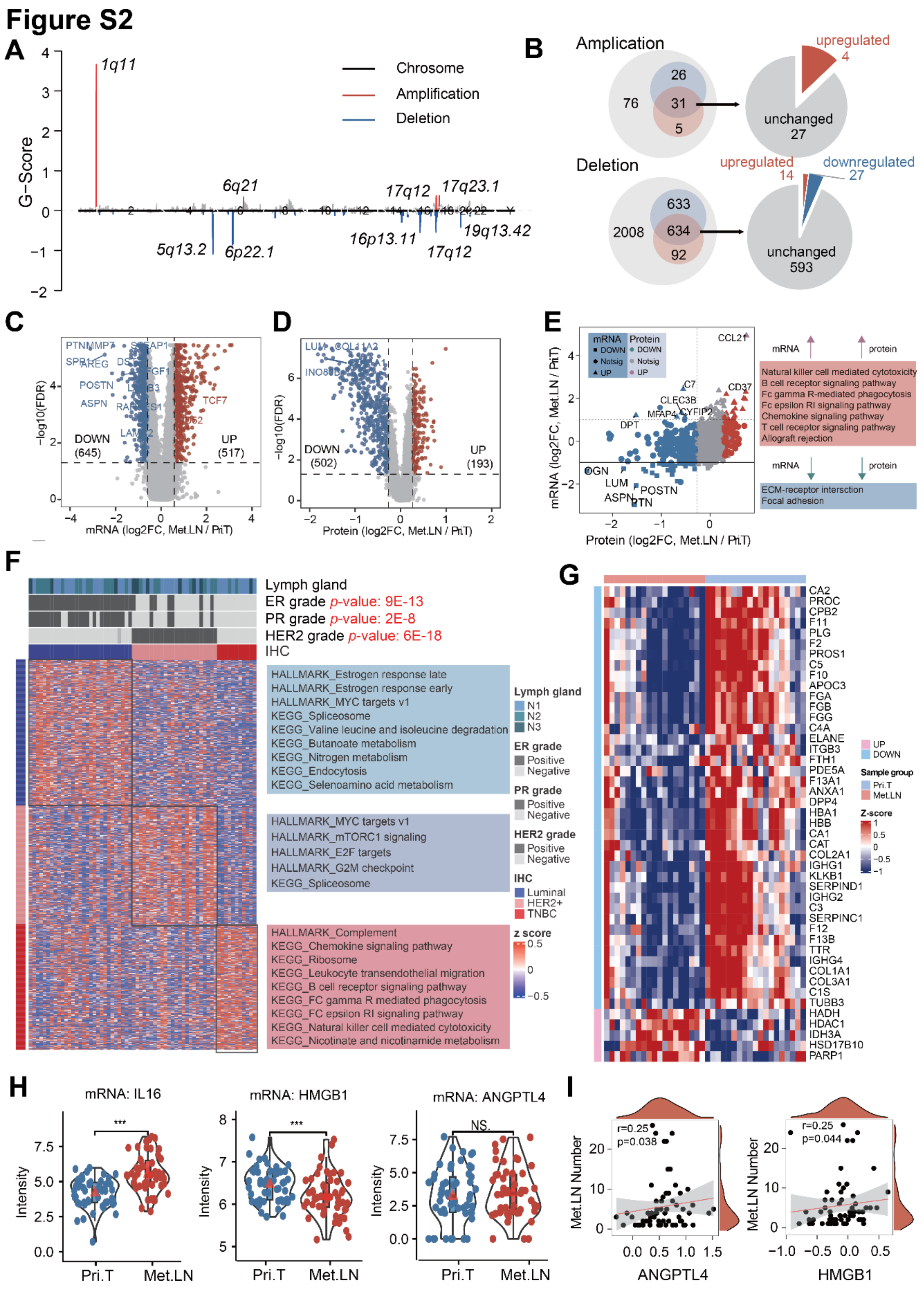

Figure S2
A
G-Score
Chrosome
Amplification
Deletion
1q11
6q21
17q12
17q23.1
5q13.2
6p22.1
16p13.11
17q12
19q13.42
B
Amplication
Deletion
upregulated
downregulated
unchanged
C
DOWN (645)
UP (517)
−log10(FDR)
mRNA (log2FC, Met.LN / Pri.T)
D
DOWN (502)
UP (193)
Protein (log2FC, Met.LN / Pri.T)
E
mRNA (log2FC, Met.LN / Pri.T)
Protein (log2FC, Met.LN / Pri.T)
Natural killer cell mediated cytotoxicity
B cell receptor signaling pathway
Fc gamma R-mediated phagocytosis
Fc epsilon RI signaling pathway
Chemokine signaling pathway
T cell receptor signaling pathway
Allograft rejection
ECM-receptor intersction
Focal adhesion
F
Lymph gland
ER grade p-value: 9E-13
PR grade p-value: 2E-8
HER2 grade p-value: 6E-18
IHC
HALLMARK_Estrogen response late
HALLMARK_Estrogen response early
HALLMARK_MYC targets v1
KEGG_Spliceosome
KEGG_Valine leucine and isoleucine degradation
KEGG_Butanoate metabolism
KEGG_Nitrogen metabolism
KEGG_Endocytosis
KEGG_Selenoamino acid metabolism
HALLMARK_MYC targets v1
HALLMARK_mTORC1 signaling
HALLMARK_E2F targets
HALLMARK_G2M checkpoint
KEGG_Spliceosome
HALLMARK_Complement
KEGG_Chemokine signaling pathway
KEGG_Ribosome
KEGG_Leukocyte transendothelial migration
KEGG_B cell receptor signaling pathway
KEGG_FC gamma R mediated phagocytosis
KEGG_FC epsilon RI signaling pathway
KEGG_Natural killer cell mediated cytotoxicity
KEGG_Nicotinate and nicotinamide metabolism
Lymph gland N1 N2 N3
ER grade Positive Negative
PR grade Positive Negative
HER2 grade Positive Negative
IHC Luminal HER2+ TNBC
z score
G
CA2 PROC CPB2 F11 PLG F2 PROS1 C5 F10 APOC3 FGA FGB FGG C4A ELANE ITGB3 FTH1 PDE5A F13A1 ANXA1 DPP4 HBA1 HBB CA1 CAT COL2A1 IGHG1 KLKB1 SERPIND1 IGHG2 C3 SERPINC1 F12 F13B TTR IGHG4 COL1A1 COL3A1 C1S TUBB3 HADH HDAC1 IDH3A HSD17B10 PARP1
UP
DOWN
Sample group Pri.T Met.LN
Z-score
H
mRNA: IL16
mRNA: HMGB1
mRNA: ANGPTL4
Intensity
Pri.T
Met.LN
I
r=0.25 p=0.038
r=0.25 p=0.044
Met.LN Number
ANGPTL4
HMGB1

Figure S2. Proteogenomic consequences of genetic alteration of Paired Metastatic Lymph Nodes and Primary Tumors, Related to Figure 2.

A. Frequencies of chromosome segment copy number amplifications (red) and deletions (blue) in Met.LNs compared to Pri.Ts.

B. Venn diagram illustrating the impact of gene amplification and deletion on protein expression.

C, D. Volcano plot depicting differentially expressed mRNAs (C) and proteins (D) between Pri.Ts and paired Met.LNs (Wilcoxon signed-rank test, Benjamini-Hochberg [BH] adjusted). Significantly up- or down-regulated genes or proteins are indicated by red or blue dots, respectively.

E. Scatterplots depicting comparison of protein (x-axis) and mRNA (y-axis) expression levels between primary Pri.Ts and paired Met.LNs, with differentially expressed genes and proteins labeled. KEGG pathway enrichment analysis was performed on genes with co-directional changes at both transcriptional and protein levels in Met.LNs compared with Pri.Ts.

F. Differentially expressed proteins in Pri.Ts compared with NATs across different IHC groups and their associated biological pathways.

G. Differentially expressed FDA-approved drug target proteins between Pri.Ts and Met.LNs, analyzed in samples with high tumor purity (⩾0.65).

H. Comparison of IL16, HMGB1, and ANGP14 mRNA levels between Pri.Ts and paired Met.LNs (Wilcoxon signed-rank test, ** represented p-value < 0.01, *** represented p-value < 0.001, NS represented p-value > 0.05).

I. Spearman correlation analysis of ANGPTL4 and HMGB1 protein expression with the number of lymph nodes metastasis using Pri.Ts and paired Met.LNs.

Figure. S3.

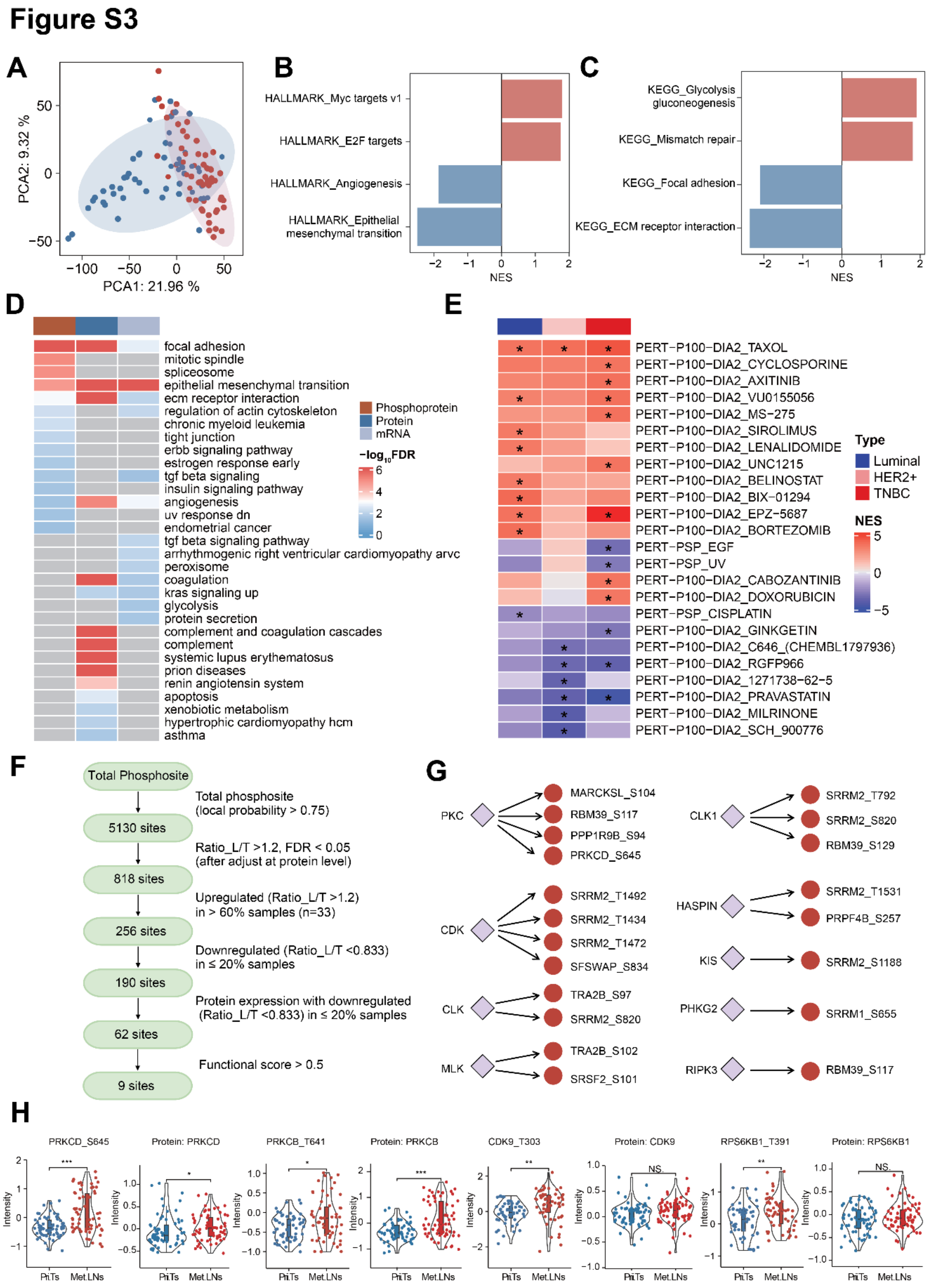
Figure S3
A
PCA2: 9.32 %
PCA1: 21.96 %
B
HALLMARK_Myc targets v1
HALLMARK_E2F targets
HALLMARK_Angiogenesis
HALLMARK_Epithelial mesenchymal transition
NES
C
KEGG_Glycolysis gluconeogenesis
KEGG_Mismatch repair
KEGG_Focal adhesion
KEGG_ECM receptor interaction
NES
D
focal adhesion
mitotic spindle
spliceosome
epithelial mesenchymal transition
ecm receptor interaction
regulation of actin cytoskeleton
chronic myeloid leukemia
tight junction
erbb signaling pathway
estrogen response early
tgf beta signaling
insulin signaling pathway
angiogenesis
uv response dn
endometrial cancer
tgf beta signaling pathway
arrhythmogenic right ventricular cardiomyopathy arvc
peroxisome
coagulation
kras signaling up
glycolysis
protein secretion
complement and coagulation cascades
complement
systemic lupus erythematosus
prion diseases
renin angiotensin system
apoptosis
xenobiotic metabolism
hypertrophic cardiomyopathy hcm
asthma
Phosphoprotein
Protein
mRNA
−log10FDR
E
PERT−P100−DIA2_TAXOL
PERT−P100−DIA2_CYCLOSPORINE
PERT−P100−DIA2_AXITINIB
PERT−P100−DIA2_VU0155056
PERT−P100−DIA2_MS−275
PERT−P100−DIA2_SIROLIMUS
PERT−P100−DIA2_LENALIDOMIDE
PERT−P100−DIA2_UNC1215
PERT−P100−DIA2_BELINOSTAT
PERT−P100−DIA2_BIX−01294
PERT−P100−DIA2_EPZ−5687
PERT−P100−DIA2_BORTEZOMIB
PERT−PSP_EGF
PERT−PSP_UV
PERT−P100−DIA2_CABOZANTINIB
PERT−P100−DIA2_DOXORUBICIN
PERT−PSP_CISPLATIN
PERT−P100−DIA2_GINKGETIN
PERT−P100−DIA2_C646_(CHEMBL1797936)
PERT−P100−DIA2_RGFP966
PERT−P100−DIA2_1271738−62−5
PERT−P100−DIA2_PRAVASTATIN
PERT−P100−DIA2_MILRINONE
PERT−P100−DIA2_SCH_900776
Type
Luminal
HER2+
TNBC
NES
F
Total Phosphosite
Total phosphosite (local probability > 0.75)
5130 sites
Ratio_L/T >1.2, FDR < 0.05 (after adjust at protein level)
818 sites
Upregulated (Ratio_L/T >1.2) in > 60% samples (n=33)
256 sites
Downregulated (Ratio_L/T <0.833) in ≤ 20% samples
190 sites
Protein expression with downregulated (Ratio_L/T <0.833) in ≤ 20% samples
62 sites
Functional score > 0.5
9 sites
G
PKC
MARCKSL_S104
RBM39_S117
PPP1R9B_S94
PRKCD_S645
CDK
SRRM2_T1492
SRRM2_T1434
SRRM2_T1472
SFSWAP_S834
CLK
TRA2B_S97
SRRM2_S820
MLK
TRA2B_S102
SRSF2_S101
CLK1
SRRM2_T792
SRRM2_S820
RBM39_S129
HASPIN
SRRM2_T1531
PRPF4B_S257
KIS
SRRM2_S1188
PHKG2
SRRM1_S655
RIPK3
RBM39_S117
H
PRKCD_S645
Protein: PRKCD
PRKCB_T641
Protein: PRKCB
CDK9_T303
Protein: CDK9
RPS6KB1_T391
Protein: RPS6KB1
Intensity
PriTs
Met.LNs
NS.

Figure S3. Phosphorylation Differences in Primary Tumors and Paired Metastatic Lymph Nodes, Related to Figure 3.

A. Principal-component analysis (PCA) plots of phosphosite in Pri.Ts (blue triangles, n = 65) and paired Met.LNs (red circles, n = 65).

B, C. Enrichment analysis using Hallmark gene sets (B) and KEGG pathways (C) of identified differentially quantified phosphorylated proteins between Pri.Ts and Met.LNs.

D. Down-regulated pathways in phosphorylation, protein expression, and transcription levels in paired Met.LNs compared with Pr.Ts stratified by subtype.

E. Heatmap showing PTM-SEA results stratified by subtype. * represented p-value < 0.05.

F. Workflow for screening key differentially expressed phosphorylation sites in Pri.Ts (n = 65) and paired Met.LNs (n = 65).

G. Diagram showing kinases with increased activity inferred from KSEA and the corresponding kinase activating sites.

H. Comparison of PRKCD, PRKCB, CDK9, and RPS6KB1 protein level and phosphorylation level between Pri.Ts and paired Met.LNs (Wilcoxon signed-rank test, n = 65, NS represented p-value > 0.05, * represented p-value < 0.05, ** represented p-value < 0.01, and *** represented p-value < 0.001).

Figure. S4.

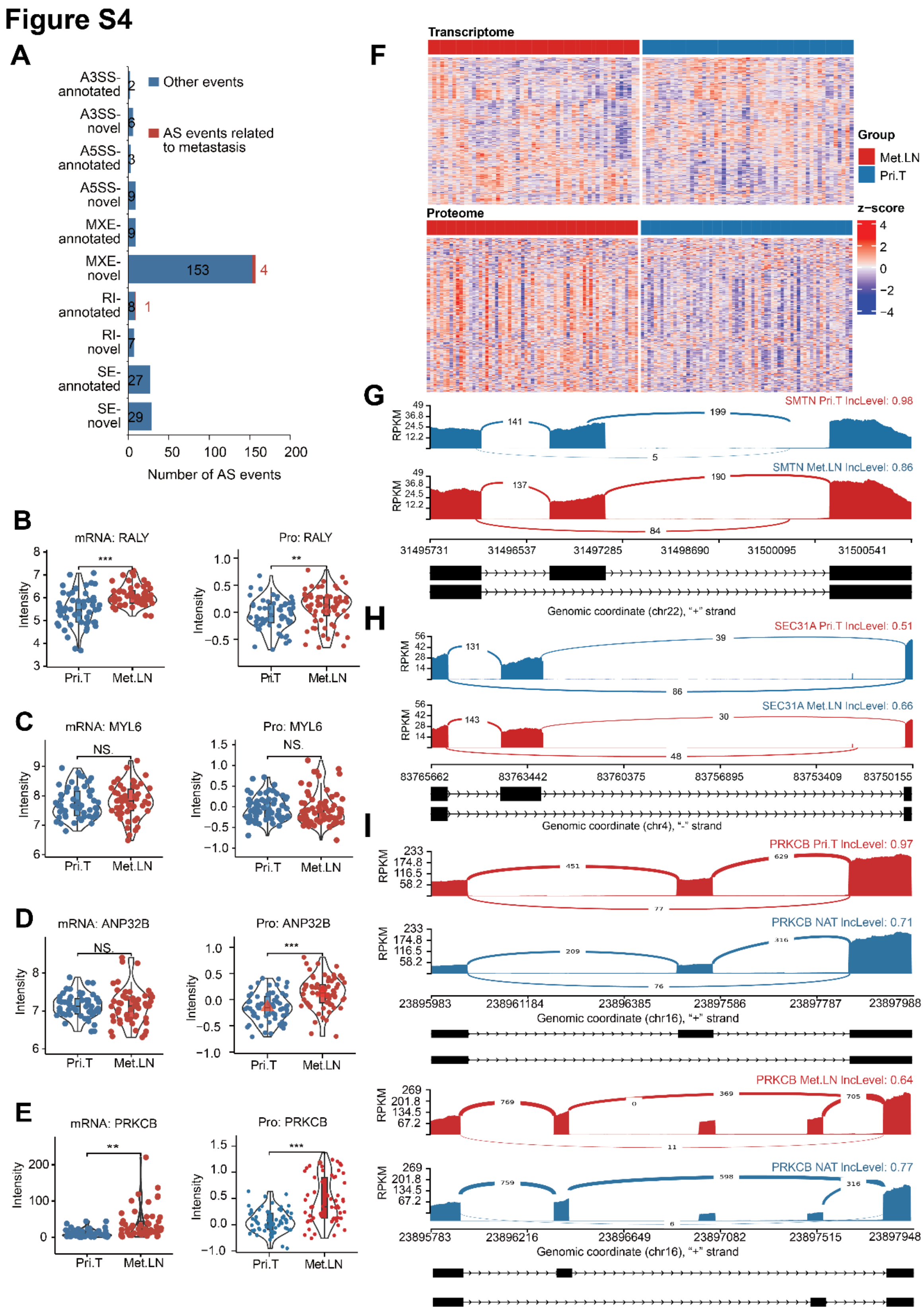

Figure S4
A
Other events
AS events related to metastasis
A3SS-annotated
A3SS-novel
A5SS-annotated
A5SS-novel
MXE-annotated
MXE-novel
RI-annotated
RI-novel
SE-annotated
SE-novel
Number of AS events
B
mRNA: RALY
Pro: RALY
C
mRNA: MYL6
Pro: MYL6
D
mRNA: ANP32B
Pro: ANP32B
E
mRNA: PRKCB
Pro: PRKCB
Intensity
Pri.T
Met.LN
F
Transcriptome
Proteome
Group
z-score
G
SMTN Pri.T IncLevel: 0.98
SMTN Met.LN IncLevel: 0.86
Genomic coordinate (chr22), "+" strand
H
SEC31A Pri.T IncLevel: 0.51
SEC31A Met.LN IncLevel: 0.66
Genomic coordinate (chr4), "-" strand
I
PRKCB Pri.T IncLevel: 0.97
PRKCB NAT IncLevel: 0.71
Genomic coordinate (chr16), "+" strand
PRKCB Met.LN IncLevel: 0.64
PRKCB NAT IncLevel: 0.77
Genomic coordinate (chr16), "+" strand
RPKM

Figure S4. Aberrant Alternative Splicing Events between Primary Tumors and Paired Metastatic Lymph Nodes, Related to Figure 4.

A. Number of significantly altered alternative splicing events between Pri.Ts and paired Met.LNs (absolute IncLevelDifference > 0.1 and FDR < 0.1). "Novel" refers to splicing events which were unannotated at previous.

B-E. Comparison of transcriptional and protein expression levels of high-frequency alterative splicing genes (*RALY* (B), *MYL6* (C), *ANP32B* (D), *PRKCB* (E)) in Pri.Ts and paried Met.LNs. (Wilcoxon sign-rank test, n = 65, NS represented *p*-value > 0.05, ** represented *p*-value < 0.01, *** represented *p*-value < 0.001)

F. A systematic comparison of splicing regulatory factors between Pri.Ts and their paired Met.LNs was conducted at both the transcriptional and protein expression levels.

G-H. Sashimiplots illustrating differentially quantified alternative splicing events occurring in *SMTN* (F), *SEC31A* (G), and *PRKCB* (H) between Pri.T and Met.LN (FDR < 0.1 and absolute IncLevelDifference > 0.1).

Figure. S5.

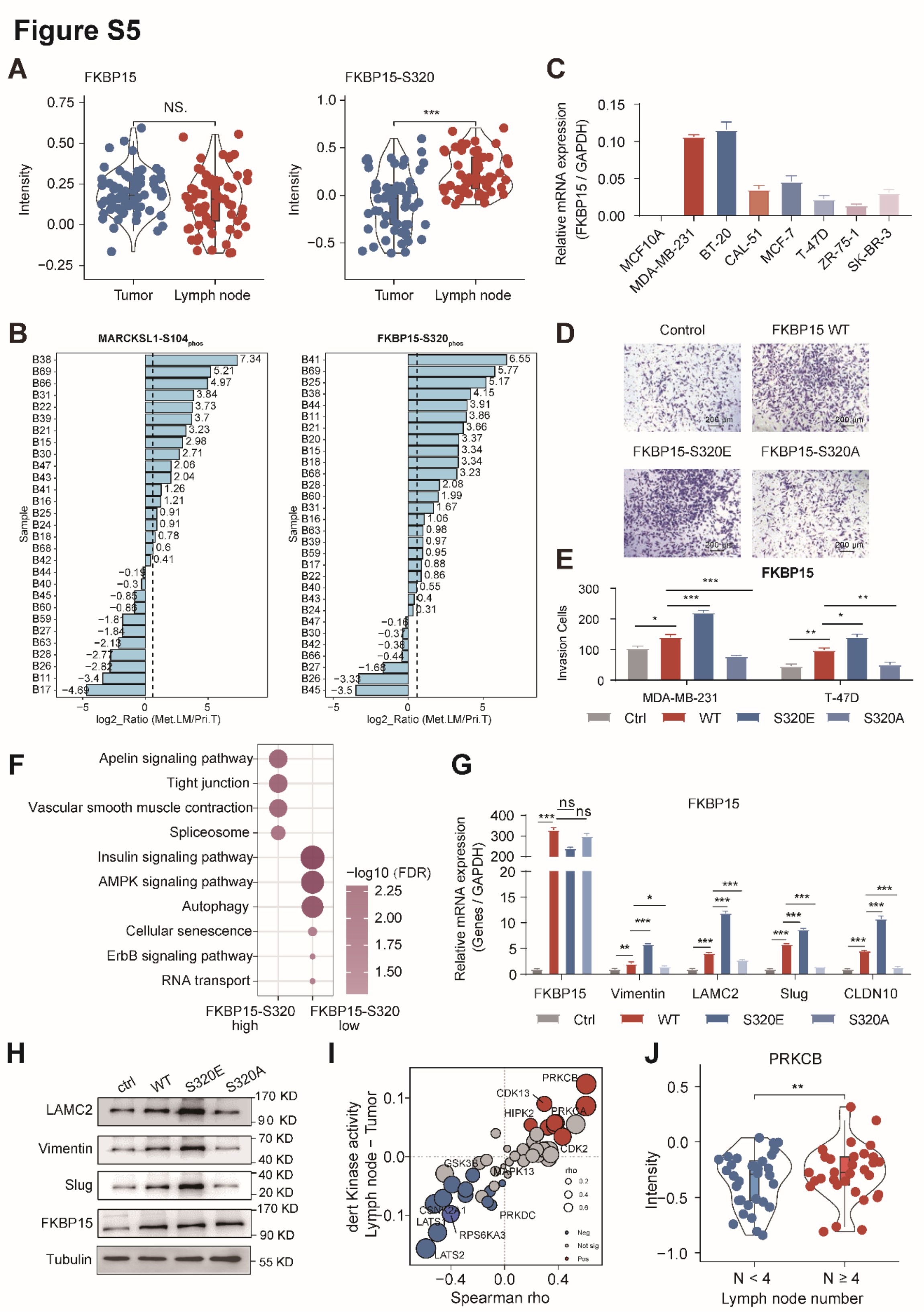

Figure S5
A
FKBP15
FKBP15-S320
NS.
***
Intensity
Tumor
Lymph node
C
Relative mRNA expression
(FKBP15 / GAPDH)
MCF10A
MDA-MB-231
BT-20
CAL-51
MCF-7
T-47D
ZR-75-1
SK-BR-3
B
MARCKSL1-S104phos
FKBP15-S320phos
Sample
log2_Ratio (Met.LM/Pri.T)
D
Control
FKBP15 WT
FKBP15-S320E
FKBP15-S320A
E
FKBP15
Invasion Cells
MDA-MB-231
T-47D
Ctrl
WT
S320E
S320A
F
Apelin signaling pathway
Tight junction
Vascular smooth muscle contraction
Spliceosome
Insulin signaling pathway
AMPK signaling pathway
Autophagy
Cellular senescence
ErbB signaling pathway
RNA transport
−log10 (FDR)
FKBP15-S320 high
FKBP15-S320 low
G
FKBP15
Relative mRNA expression
(Genes / GAPDH)
FKBP15
Vimentin
LAMC2
Slug
CLDN10
H
ctrl
WT
S320E
S320A
LAMC2
Vimentin
Slug
FKBP15
Tubulin
170 KD
90 KD
70 KD
40 KD
20 KD
55 KD
I
dert Kinase activity
Lymph node − Tumor
Spearman rho
PRKCB
CDK13
HIPK2
PRKCA
CDK2
GSK3B
MAPK13
PRKDC
RPS6KA3
LATS1
LATS2
J
PRKCB
**
Intensity
N < 4
N ≥ 4
Lymph node number

Figure S5. Identification and Validation of Phosphorylation Metastasis Biomarkers, Related to Figure 5.

A. Comparison of FKBP15 protein and phosphorylation levels between Pri.Ts and paired Met.LNs (Wilcoxon signed-rank test, n = 65, NS represented *p*-value > 0.05 and *** represented *p*-value < 0.001).

B. Quantitative comparison of the phosphorylation levels at MARCKSL1-S104 and FKBP15-S320 sites in tumor tissues and paired lymph node tissues was conducted using PRM technology.

C. Analysis of *FKBP15* mRNA expression levels performed by real-time qPCR in breast cancer cell lines.

D, E. The impacts of FKBP15-S320 WT or mutant overexpression on breast cancer cell migration. Trans-well migration assays (D) of indicated cells were measured. Data (E) are represented as mean ± SEM (Student's t test), **p*-value < 0.05, ***p*-value < 0.01, ****p*-value < 0.001. (Scale bars, 50 mm.)

F. Representative KEGG pathways comparing high FKBP15-S320 with low FKBP15-S320 groups in Pri.Ts.

G. Real-time qPCR validation of representative FKBP15-S320 target genes in cells overexpressing WT versus S320E or S320A mutants. Data are shown as mean ± SEM (Student's t test), * *p*-value < 0.05, ** *p*-value < 0.01, *** *p*-value < 0.001.

H. Western blot validation of FKBP15 target genes in CAL-51 cells overexpressing FKBP15-WT compared to S320E or S320A mutants.

I. Dot plot showing the Spearman correlation between kinase activity and phosphorylation level of FKBP15 S320 and the difference of kinase activity assessed using ssGSEA between Pri.Ts and Met.LNs.

J. Comparison of PRKCB protein expression levels between Pri.Ts samples with low ($n \leq 3$) and high ($n \geq 4$) numbers of lymph nodes metastasis (Wilcoxon rank-sum test, ** represented *p*-value < 0.01).

Figure. S6.

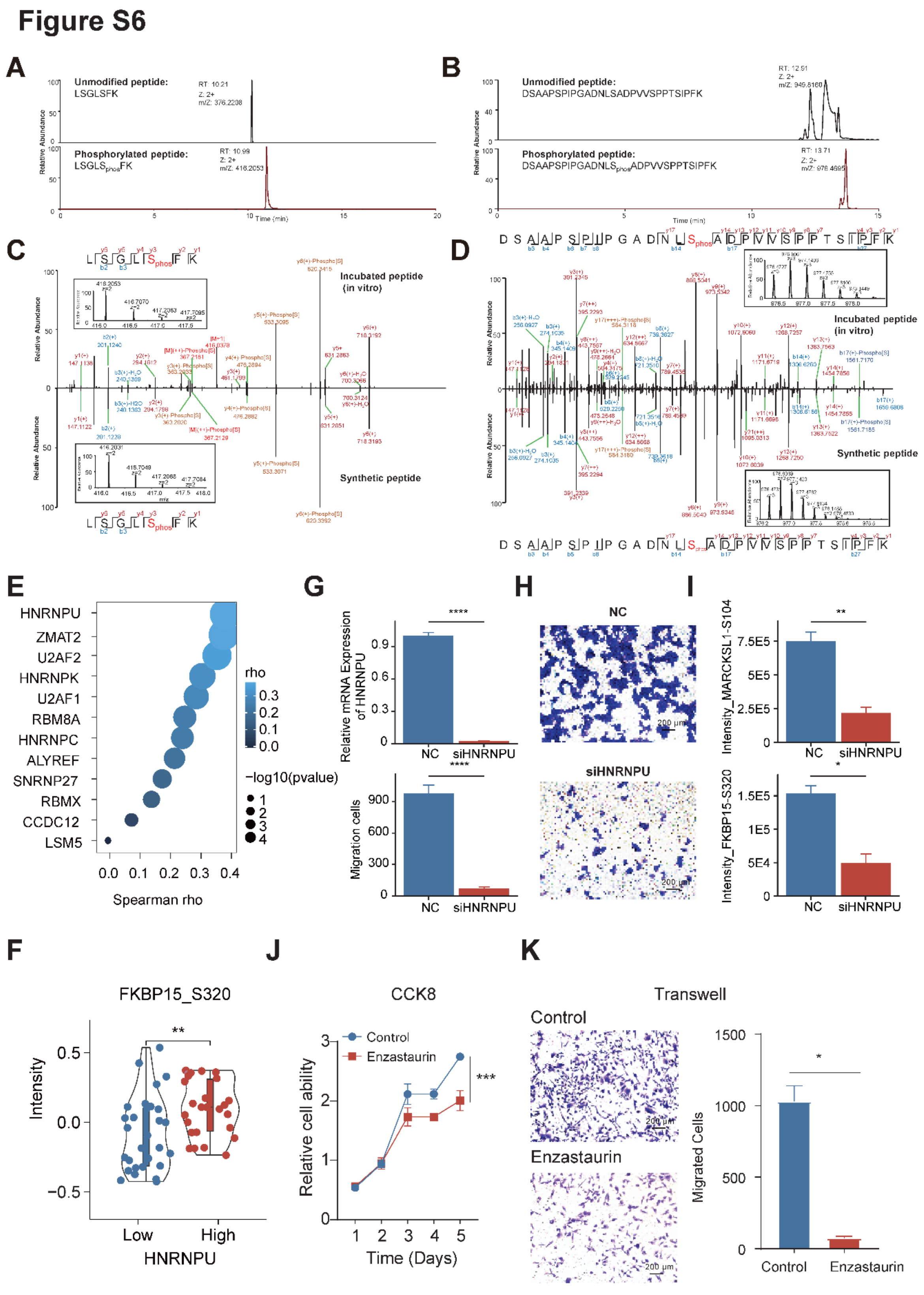

Figure S6
A
Unmodified peptide:
LSGLSFK
Phosphorylated peptide:
Relative Abundance
Time (min)
B
Unmodified peptide:
DSAAPSPIPGADNLSADPVVSPPTSIPFK
Phosphorylated peptide:
C
Incubated peptide (in vitro)
Synthetic peptide
D
Incubated peptide (in vitro)
Synthetic peptide
E
HNRNPU
ZMAT2
U2AF2
HNRNPK
U2AF1
RBM8A
HNRNPC
ALYREF
SNRNP27
RBMX
CCDC12
LSM5
rho
−log10(pvalue)
Spearman rho
G
Relative mRNA Expression of HNRNPU
Migration cells
NC
siHNRNPU
H
NC
siHNRNPU
I
Intensity_MARCKSL1-S104
Intensity_FKBP15-S320
F
FKBP15_S320
Intensity
Low
High
HNRNPU
J
CCK8
Control
Enzastaurin
Relative cell ability
Time (Days)
K
Transwell
Control
Enzastaurin
Migrated Cells

Figure S6. Identification and Validation of Phosphorylation-Based Metastasis Biomarkers Using Synthetic Peptide and Cell Migration Analyses , Related to Figure 5.

A. Comparison of extracted ion chromatographic peaks between the original synthetic peptide (LSGLSFK, a peptide containing the S104 site of MARCKSL1) and the peptide generated from in vitro enzymatic assays.

B. Comparison of extracted ion chromatographic peaks between the original synthetic peptide (DSAAPSPIPGADNLSADPVVSPPTSIPFK, a peptide containing the S320 site of FKBP15) and the peptide generated from in vitro enzymatic assays.

C. MS/MS spectra of the modified peptide (LSGLS[phos]FK) derived from the in vitro kinase assay (top panel) and synthesized phosphorylated peptide standards (bottom panel).

D. MS/MS spectra of the modified peptide (DSAAPSPIPGADNLS[phos]ADPVVSPPTSIPFK) derived from the in vitro kinase assay (top panel) and synthesized phosphorylated peptide standards (bottom panel)..

E. Spearman correlation between splicing factors expression level and phosphorylation level of MARCKSL1-S104.

F. Comparison of FKBP15-S320 phosphorylation levels between samples with high versus low protein expression levels of HNRNPU (Wilcoxon rank-sum test, ** represented p-value < 0.01).

G. Assessment of *HNRNPU* mRNA expression levels in *HNRNPU*-knockdown MCF7 cells (Upper panels, Student's t-test, **** represented p-value < 0.0001); Quotative assessment of cell migration activity in *HNRNPU*-knockdown and control MCF7 cells (Bottome panels, Student's t-test, **** represented p-value < 0.0001).

H. Evaluation of cell migration capacity in MCF7 cells following *HNRNPU* knockout;

I. Comparative analysis of phosphorylation levels at MARCKSL1-Ser104 and FKBP15-Ser320 between *HNRNPU*-knockdown and wild-type control cells (Student's t-test, * represented p-value < 0.05, ** represented p-value < 0.01)

J. The CCK8 analysis was conducted to evaluate the cell ability after of targeted inhibition of PRKCB activity on MDA-MB-231 cancer cells (*** represented p-value < 0.001).

K. Cell migration assays confirm the effect of targeted PRKCB activity inhibition on the migratory behavior of MDA-MB-231 cancer cells (Student's t-test, * represented p-value $<$ 0.05).

Figure. S7.

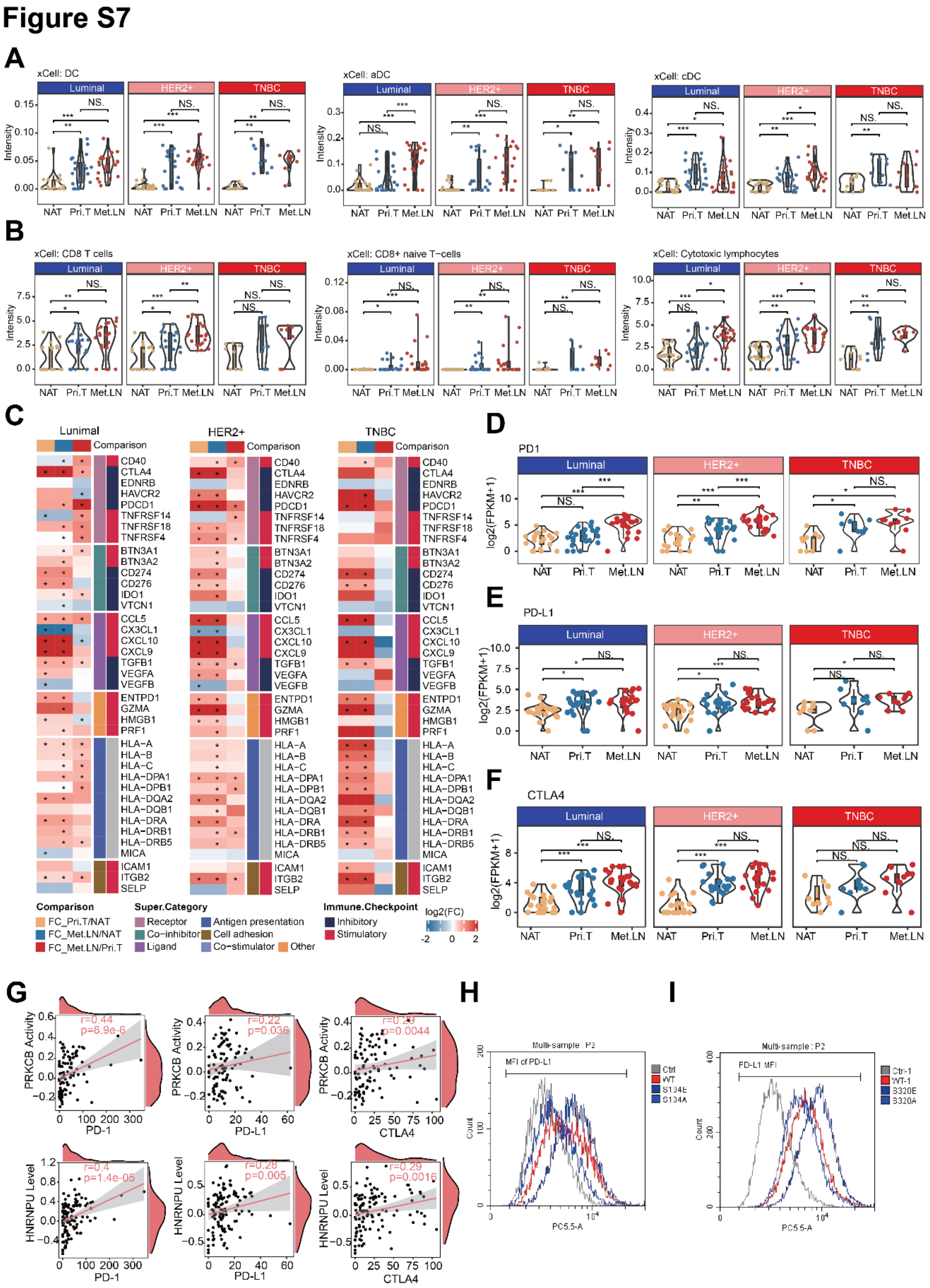
Figure S7
A
xCell: DC
xCell: aDC
xCell: cDC
Luminal
HER2+
TNBC
Intensity
NAT
Pri.T
Met.LN
B
xCell: CD8 T cells
xCell: CD8+ naive T−cells
xCell: Cytotoxic lymphocytes
C
Comparison
CD40
CTLA4
EDNRB
HAVCR2
PDCD1
TNFRSF14
TNFRSF18
TNFRSF4
BTN3A1
BTN3A2
CD274
CD276
IDO1
VTCN1
CCL5
CX3CL1
CXCL10
CXCL9
TGFB1
VEGFA
VEGFB
ENTPD1
GZMA
HMGB1
PRF1
HLA−A
HLA−B
HLA−C
HLA−DPA1
HLA−DPB1
HLA−DQA2
HLA−DQB1
HLA−DRA
HLA−DRB1
HLA−DRB5
MICA
ICAM1
ITGB2
SELP
Comparison
FC_Pri.T/NAT
FC_Met.LN/NAT
FC_Met.LN/Pri.T
Super.Category
Receptor
Co−inhibitor
Ligand
Antigen presentation
Cell adhesion
Co−stimulator
Other
Immune.Checkpoint
Inhibitory
Stimulatory
log2(FC)
D
PD1
log2(FPKM+1)
E
PD-L1
F
CTLA4
G
PRKCB Activity
HNRNPU Level
PD-1
PD-L1
CTLA4
H
Multi-sample : P2
MFI of PD-L1
Ctrl
WT
S104E
S104A
Count
PC5.5-A
I
Multi-sample : P2
PD-L1 MFI
Ctr-1
WT-1
S320E
S320A

Figure S7. Immune Characteristics of Primary Tumors and Paired Metastatic Lymph Nodes, Related to Figure 6.

A. Comparison of DC, aDC and cDC with xCell-derived immune cell signatures between NATs, Pri.Ts and Met.LNs across different IHC subtypes (**p*-value < 0.05, ** *p*-value < 0.01, *** *p*-value < 0.001, adjusted *p*-value from Wilcoxon signed-rank test). DC, dendritic cell; aDC, activated dendritic; cDC, classical dendritic cell.

B. Comparison of $CD8^+$ T, naïve T and cytotoxic T cells with xCell-derived immune cell signatures between NATs, Pri.Ts and Met.LNs across different IHC subtypes (**p*-value < 0.05, ** *p*-value < 0.01, *** *p*-value < 0.001, adjusted p-value from Wilcoxon signed-rank test).

C. Heatmap illustrating differentially expressed proteins in different IHC subtype between NATs, Pri.Ts and Met.LNs involved in MHC molecules, stimulatory and inhibitory immune modulators (**p*-value < 0.05, ** *p*-value < 0.01, *** *p*-value < 0.001, Wilcoxon signed-rank test).

D-F. Comparison of PD1 (D), PD-L1 (E) and CTLA4 (F) protein expression between NATs, Pri.Ts and Met.LNs across different IHC subtypes (Wilcoxon rank-sum test, ns represented *p*-value > 0.05, **p*-value < 0.05, ** *p*-value < 0.01, *** *p*-value < 0.001).

G. Scatterplot showing Spearman's correlation of MARCKSL1-S104 phosphorylation levels and PD-1, PD-L1, and CTLA4 expression in all samples.

H-I. PD-L1 expression in *MARCKSL1* wild-type and mutated cells (H), as well as in *FKBP15* wild-type and mutated cells (I) was analyzed by flow cytometry and the representative images are shown.

Materials and methods

Clinical Sample Acquisition

Patients diagnosed with malignant breast cancer in Fudan University Shanghai Cancer Center (FUSCC) and willing to participate in the present study provided written informed consent. This study was approved by the Ethics Committee of FUSCC (Number: FUSCC-IACUC-S2024-0462). In total, 65 primary tumors, paired non-cancerous adjacent breast tissues, and paired metastatic lymph nodes were sequenced and utilized in further analyses. All primary tumors were confirmed as invasive carcinoma and metastatic status of lymph nodes were confirmed by pathologists through both intraoperative frozen section and the paraffin section after surgery. Non-cancerous adjacent tissues were acquired as defined as tissues located adjacent to the tumor but 2 cm outside the visible abnormalities. The percentage of tumor cells was confirmed to be 50% or more in all the primary tumors and paired metastatic lymph nodes. Clinical characteristics and tumor clinicopathologic information are summarized in **Table S1**.

Chemicals and Reagents

Chemicals purchased included Protease inhibitor (06538282001, Roche); Phosphatase inhibitor (04906837001, Roche); Urea (U0631, Sigma Aldrich); Ammonium bicarbonate (09830, Sigma Aldrich); Dithiothreitol (43815, Sigma Aldrich); Iodoacetamide (16125, Sigma Aldrich); Trypsin (HLS TRY001C, Hualishi); TMT tags (90111, Thermo Scientific); TMT11-131C (A37724, Thermo Scientific); Ammonium hydroxide solution (221228, Merck); Triethylammonium bicarbonate buffer (T7408, Sigma Aldrich); Acetonitrile (10014108, Sinopharm); Titanium (IV) sulfate (20044428, Sinopharm); Trifluoroacetic acid (T6508, Sigma Aldrich); NaCl (10019318, Sinopharm); Trifluoroacetic acid (T6508, Sigma Aldrich), Formic acid (56302, Honeywell Fluka), and Enzastaurin (HY-10342, MedChemExpress).

Tissue Sample Preparation

The excised tissue samples obtained during surgical procedures are initially rinsed with pre-cooled physiological saline to eliminate any residual blood from the tissue surface. Subsequently, the tissues were rapidly frozen in liquid nitrogen for preservation. The frozen tissues were then transferred to be stored at -80 °C until used for various applications, such as protein and DNA extraction. The tissues used for RNA extraction were immersed in RNAlater Stabilization Solution (AM7020, Thermo Fisher Scientific) before the extraction process.

Protein Extraction

The collected tissue samples were stored in a -80 °C freezer prior to use. After thawing on ice, the tissues were washed with pre-cooled PBS solution to thoroughly remove residual blood and impurities. The tissues were lysis and protein extraction were using 8 M urea lysis buffer (8 M urea, 100 mM ammonium bicarbonate, 1 × protease inhibitor, 4 × phosphatase inhibitor, pH = 8.0). The tissue samples were lysed with about 10 times the volume of urea lysis buffer. Then the tissues were sonicated on ice for 5 min with 2 s on and 3 s off. Then the tissue lysis was centrifuged under 4 ºC with 20,000 g for 10 min. The supernatants were then collected and the precipitated tissue debris were removed. The extracted protein concentration was measured by using BCA protein assay (P0012, Beyotime).

Protein Reduction, Alkylation and Tryptic Digestion

The extracted proteins were reduced by with 5 mM dithiothreitol (DTT) for 30 min at 56 °C, and then alkylated with 15 mM iodoacetamide for 30 min in darkness at room temperature. Finally, the alkylation was quenched by adding DTT with 20 mM for 30 min at room temperature. The urea concentration of the protein solution was

subsequently diluted into lower than 2 M by adding four volumes of 100 mM ammonium bicarbonate. The in-solution tryptic digestion was conducted with a trypsin: protein ratio of 1:50 (w/w) at 37 °C overnight. To ensure complete digestion, an additional round of digestion was carried out for another 4 hours using the trypsin: protein ratio of 1:100 (w/w) at 37 °C. The tryptic peptides were desalted using SepPak tC_{18} cartridges (WAT054960, Waters, MA), followed by vacuum-dried via Speed Vac system (SPD120-230, Thermo Scientific) and subsequent storage at −80°C before analysis.

11-plex TMT Labeling

The desalted peptides were labeled with TMT tags according to the instruction. Briefly, the dried desalted peptides were dissolved in 200 μL 50mM triethylammonium bicarbonate buffer (TEAB). All samples were separated into 12 groups with 10 samples in each group. Each sample group were labeled with a 11-plex TMT reagent (Thermo fisher, China). For each batch of 11-plex labeling, the TMT-131C channel was reserved for the internal reference sample, which was derived from the same amount peptide mixture of all tissue samples. Each plex TMT reagent was dissolved in acetonitrile (ACN) and added into the related peptide sample with the peptide: TMT ratio of 1:8 (w/w). The reactions were incubated at room temperature for one hour and subsequently quenched using a 5% Ammonium hydroxide solution. All the labeling efficiency was ensured higher than 95%. In each group, all 11 samples were mixed with a 1:1 ratio and then desalted by SepPak C_{18} cartridges.

High-pH HPLC Fractionation

The TMT-labeled peptides were subsequently fractionated using high-pH reverse phase liquid chromatography with a 90-minute gradient at a flow rate of 1 mL/min. A total of 3.3 mg of the labeled peptide sample was dissolved in buffer A (98% H_2O, 2% acetonitrile, pH 9.5) and loaded onto an Xbridge C_{18} column (4.6 mm × 250 mm, pore size: 130 Å, particle size:3.5 μm, 186003117, Waters) equipped on Agilent 1200 HPLC system (Agilent). An increasing gradient of buffer B (98% acetonitrile, 2% H_2O, pH 9.5) was applied from 0% to 95% over a duration of 90 minutes for elution purposes. For phosphorylation enrichment analysis, approximately 95% of the peptide samples were combined into twelve fractions while about a remaining amount of approximately five percent peptides were pooled into twenty-four fractions for proteome profiling analysis. Subsequently, these samples were dried using a Speed Vac.

Enrichment of the Phosphopeptide

The phosphopeptide enrichment was performed using a Ti^{4+}-IMAC-based phosphopeptide enrichment method. Initially, microspheres were mixed with Titanium (IV) sulfate and incubated overnight at room temperature. Subsequently, Ti^{4+}-IMAC-loaded spin tips were assembled and pre-equilibrated using the loading buffer (80% ACN and 6% trifluoroacetic acid (TFA)). Each of the 12 fractions in every group was dissolved in 250 μL of loading buffer and loaded onto the tip. After centrifugation at 100 g, the flowthrough was collected and reloaded onto the spin tips. The bound phosphorylated samples were subsequently washed with washing buffer 1 (50% ACN, 6% TFA containing 200 mmol/L NaCl) and washing buffer 2 (30% ACN, 0.1% TFA), respectively, to remove non-phosphorylated peptides through an additional round of centrifugation at a speed of 100 g each time. Finally, the bound phosphorylated peptides were sequentially eluted using elution buffer A (10% $NH_3 \cdot H_2O$) followed by elution buffer B (10% $NH_3 \cdot H_2O$, 40% ACN). The samples were dried in a Speed Vac for further utilization.

Nano-LC-MS/MS

For proteome profiling analysis, the fractionated peptides were detected by a nanoflow

HPLC Easy-nLC 1200 system (Thermo Fisher Scientific) tandem with Q Exactive HF-X mass spectrometer (Thermo Fisher Scientific). Initially, the peptides were resuspended in mobile phase buffer A (0.1% formic acid and 2% acetonitrile in water) and then loaded onto a home-made C_{18} nano-capillary analytical column (75 μm × 220 mm) packed with C_{18} resin (particle size 1.9 μm, Pore Size 120Å, ReproSil-Pur 120 C18-AQ, Dr. Maisch) through the autosampler equipped on Easy-nLC 1200 system. A column oven (SCO-2110 Pro, Shanghai EasyMass Spectrometry Co.) was used to heat the capillary column with 60°C. A 70-minute LC gradient with a consistent flow rate of 300 nL/min was used. The elution gradient consisted of the following steps: 6%-30% buffer B (0.1% formic acid, 90% acetonitrile in water) in 0-57 min; 30%-45% buffer B in 4 min; 45%-80% buffer B in 4 min and 80% buffer B in 5 min. The eluted peptides were ionized using a nano-spray ion source equipped on the Q Exactive HF-X mass spectrometer under a voltage of 2kV. The ion transfer tube was maintained at a temperature of 300°C. Data-dependent acquisition mode was performed using Xcalibur software. For the MS1, ions were detected by orbitrap mass analyzer at a resolution of 60,000 (m/z 200). The scan range was m/z 450-1,700. The automatic gain control (AGC) target was set to 3 X 10^6, and the maximal ion injection time (IT) was set at 45 ms. For MS2, the top 12 high-abundance MS1 ions were selected for fragmentation into MS/MS fragments and subsequently detected by orbitrap at the resolution of 45,000 (m/z 200). The normalized collision energy (NCE) for higher energy collision dissociation (HCD) was set to 32%. Precursor charge state screening was enabled to reject unassigned ions with charges of 1+, 6+-8+, and >8+. The fixed first mass of the MS2 spectrum was set at 100.0 m/z, and the scan range was from 200-2,000 m/z. The AGC target for this setting was adjusted to be at 1 X 10^5, while the maximal ion injection time remained at a value of 30 ms. An isolation window of size 0.8 m/z was used, and the dynamic exclusion was set as 30 s.

For phosphoproteomic analysis, 0.1% formic acid was utilized for solubilizing the enriched peptides. Half of the peptides from each fraction were separated using a nanoflow HPLC Easy-nLC 1200 system (Thermo Fisher Scientific) with a 95-minute LC gradient at a flow rate of 300 nL/min. The temperature of the homemade C_{18} column was maintained at 60 °C using a column heater. The gradient consisted of the following steps: starting from 3% buffer B and increasing to 7% B in one minute, followed by an increase to 28% B over a period of 69 minutes, then reaching 61% B in ten minutes, further increasing to 90% B within one minute, maintaining it for four minutes, decreasing to 50% B in one minute and finally holding it for nine minutes. Subsequently, the peptides were subjected to analysis on a Q Exactive HF-X mass spectrometer (Thermo Fisher Scientific). The electrospray voltage was set to 2,200 V in positive ion mode, while maintaining the ion transfer tube temperature at 300 °C. For MS1, the ions were detected by orbitrap analyzer with a resolution of 60,000 (at m/z 200) within an m/z range of 350-1,800. The AGC target was set to 3 X 10^6 and the maximal ion injection time was set to 50 ms. For MS2, the top 10 high-abundance MS1 ions with charge states of +2 ~ +5 were selected for fragmentation into MS/MS fragments and subsequently detected by orbitrap at the resolution of 45,000 (m/z 200). The HCD collision energy was set at 32%. The AGC target for MS/MS analysis was adjusted to be at least 1 X 10^5 and the maximal ion injection time increased to105 ms. The fixed first mass of the MS2 spectrum was set at 110.0 m/z. The isolation window was adjusted to 0.8 m/z, while the dynamic exclusion time was set to 20 seconds.

Parallel Reaction Monitoring (PRM) Analysis

Equal amounts (2 μg per sample) of tryptic-digested proteome samples from tumor tissues and metastatic lymph nodes were analyzed using an Orbitrap Exploris 480 mass

spectrometer (Thermo Fisher Scientific) coupled with a Vanquish Neo nano-liquid chromatography system. Peptides were separated on a home-packed C_{18} capillary column using a 35-minute gradient elution at a flow rate of 300 nL/min. The column temperature was maintained at 60 °C with a column heater. The gradient was as follows: initial hold at 1% buffer B for 2 minutes, linear increase to 20% B over 12 minutes, further ramp to 45% B in 14 minutes, sharp increase to 90% B within 2 minutes, and a final hold at 90% B for 5 minutes.

Mass spectrometric analysis was performed in parallel reaction monitoring (PRM) mode with an inclusion list containing the precursor ions of target peptides. Electrospray ionization (ESI) was operated in positive ion mode with a spray voltage of 2,200 V, and the ion transfer tube temperature was set to 300 °C. For full scan, ions were detected in the Orbitrap analyzer over an m/z range of 350–1,800 with a resolution of 60,000 (at m/z 200). The automatic gain control (AGC) target was set to 3×10^6, and the maximum ion injection time was 50 ms. For PRM scans, target precursor ions were isolated with a 0.8 m/z window and fragmented via higher-energy collisional dissociation (HCD) at 32% collision energy. MS/MS fragments were detected in the Orbitrap analyzer at a resolution of 45,000 (at m/z 200). The AGC target for MS/MS was set to at least 1×10^5, with a maximum ion injection time of 105 ms. The fixed first mass of MS2 spectra was set to 110.0 m/z. The intensity of target ions in each sample was extracted and quantified using Xcalibur software (Thermo Fisher Scientific).

Database Searching of Proteomic and Phosphoproteomic Data

The raw data for proteome profiling and phosphoproteome profiling were analyzed using MaxQuant (version 2.0.1.0, https://www.maxquant.org/) against the UniProt human proteome (05/10/2020, https://www.uniprot.org/). TMT 11-plex-based MS2 reporter ion was chosen with the precursor intensity fraction (PIF) threshold set at 0.75. The correction factors for TMT regents were imported into MaxQuant. All other parameters were kept as default settings. The carbamidomethyl cysteine was designated as a fixed modification, while methionine oxidation and protein N-terminal acetylation were considered variable modifications. Additionally, phospho (STY) was selected as a variable modification for the analysis of phosphoproteomic data. Trypsin/P was employed as the protease enzyme. A maximum of 5 modifications per peptide were allowed. The maximum number of missed cleavage sites was set at 2. The first search tolerance and main search tolerance were defined as 20 ppm and 4.5 ppm, respectively. Peptide, protein, and site false discovery rates (FDR) were all maintained below 0.01 threshold level.

WES Analysis

Total DNA from each tissue sample were extracted by using QIAamp DNA mini Kit (51306, QIAGEN, Germany) following the manufacturer's instructions. The concentration of DNA was determined by using the Qubit DNA Assay Kit. For DNA sequencing, a total of 0.6 mg genomic DNA per sample was utilized.

The exome sequences were efficiently enriched from 0.4 µg genomic DNA using Agilent liquid capture system (Agilent SureSelect Human All Exon V6) following the manufacturer's protocol. Firstly, qualified genomic DNA was randomly fragmented to an average size of 180-280 bp by Covaris S220 sonicator. Remaining overhangs were converted into blunt ends via exonuclease polymerase activities. Secondly, DNA fragments were end repaired and phosphorylated, followed by A-tailing and ligation at the 3'ends with paired-end adaptors. The DNA fragments with ligated adapter

molecules on both ends were selectively enriched through a PCR reaction. Subsequently, the libraries hybridize with liquid phase with biotin labeled probe, then use magnetic beads with streptomycin to capture the exons of genes. The captured libraries were enriched in a PCR reaction to add index tags to prepare for sequencing. Products were purified using AMPure XP system (Beckman Coulter, Beverly, USA), DNA concentration was measured by Qubit®3.0 Flurometer (Invitrogen, USA), libraries were analyzed for size distribution by NGS3K/Caliper and quantified by real-time PCR (3 nM). Finally, the DNA library were sequenced on Illumina for paired end 150 bp reads.

The raw sequencing reads were filtered and quality assessed by fastp (version 0.23.4). Then the clean reads were aligned to the human reference genome (hg38) using bwa mem. The resulting BAM files were further processed by samtools for sorting, indexing, and marking duplicate. Somatic SNV and Somatic INDEL analyses were performed with muTect and Strelka, utilizing paired tumor and normal samples or paired lymph node and normal samples. Variant annotation was conducted using VEP (GRCh38 release 109), followed by file format conversion with vcf2maf. Somatic CNV was analyzed with CNVkit (version 0.9.6) for paired tumor-normal samples or paired lymph node-normal samples and the results were further analyzed with Gistic2 (version 2.0) with the following parameters: -ta 0.2 -td 0.2 -js 100 -broad 1 -brlen 0.7 -conf 0.95 -genegistic 1 -savegene 1. The Gistic2 results were visualized using maftools and analyzed within R (v4.1.0).

RNA Extraction and RNA-Seq Data Analysis

The total RNA was extracted with trizol（15596026, Ambion/Invitrogen, USA）and the integrity of the RNA were assessed using the RNA Nano 6000 Assay Kit of the Bioanalyzer 2100 system (Agilent Technologies, CA, USA). Total RNA was then used as input material for the RNA sample preparations. Briefly, mRNA was purified from total RNA by using poly-T oligo-attached magnetic beads. Fragmentation of mRNA was carried out using divalent cations under elevated temperature in First Strand Synthesis Reaction Buffer (5X). The first strand cDNA was synthesized using random hexamer primer followed by degradation of RNA. The second strand cDNA synthesis was subsequently performed and remaining overhangs were converted into blunt ends. After adenylation of 3’ ends of DNA fragments, adaptors with hairpin loop structure were ligated to prepare for hybridization. To selectively isolate cDNA fragments rangeing from 370~420 bp in length, the library fragments were purified with AMPure XP system (Beckman Coulter, Beverly, USA). Subsequently, the PCR amplification was performed and the resulting PCR products were purified by using AMPure XP beads to obtain the final library. After the construction of the library, the library was initially quantified by Qubit2.0 Fluorometer, then diluted to 1.5ng/ul. The insert size of the library was detected by Agilent 2100 bioanalyzer. After insert size meets the expectation, qRT-PCR was used to accurately quantify the effective concentration of the library (the effective concentration of the library was higher than that of 2nM) to ensure the quality of the library.

The raw reads were filtered with fastp (version 0.19.7) and the clean reads were aligned to reference genome with HISAT2 (version 2.0.5). The BAM files were further quantified with featureCounts (version 1.5.0-p3) to generate the FPKM quantification results. The raw quantification results were filtered for protein coding gene and proteins with total FPKM as 0 were removed. The obtained quantification results were then upper-quantile normalized and log transformed.

Cell Lines and Cell Culture

Human breast cancer cell line MDA-MB 231, MCF7 and T-47D purchased from ATCC

were cultured in RPMI 1640 or DMEM medium (C11875500BT/C11995500BT, Gibico) supplemented with 10% fetal bovine serum (FBS, 10099141C, Gibco) and 1% penicillin/streptornycin (P/S, C100C5, NCM Biotech, Shanghai). All of the cell lines were tested and authenticated. All cells were cultured at 37 C in a humidified atmosphere containing 5% CO2. The cell lines were mycoplasma-free and authenticated by PCR analysis monthly, and all cell lines were performed the STR authentication,

Western Blotting

For sample preparation, total protein in cells was extracted and boiled in SDS loadingbuffer, then protein lysates were separated by 10% sDS-PAGE, transferred onto a Immobilon PVDF Transfer Membrane (IPVH00010, Millipore) and probed with specific primary antibodies. The detailed information about antibodies were shown: Vimentin (D21H3) XP® Rabbit Monoclonal Antibody (5741T, CST; Dilution, 1:1000), Slug (C19G7) Rabbit Monoclonal Antibody (9585T, CST; Dilution, 1:1000), MARCKSL1 Rabbit Monoclonal Antibody (A22957, ABclonal; Dilution, 1:2000); KBP15 Rabbit Polyclonal Antibody (PA5-144010, Thermo; Dilution, 1:2000); Anti-β-Tubulin Mouse Monoclonal Antibody (M20105, TRAN; Dilution, 1:1000). HRP substrate Luminol Reagent (23356A4, Millipore) was used to detect HRP-conjugated secondary with an Image Quant LAS 4000 mini-imaging system (GE, Fairfield). All experiments were repeated more than 3 times.

Invasion Assay

Twenty thousand cells were seeded in the8.0 μm pore size (353097, FALCON) chambers coated with Matrigel Matrix (354234, Corning Life Sciences) and placed into 24-well culture plate. After 36 hours, cells in lower chambers were fixed with formalin and stained with crystal violet. The invaded cells were photographed by microscope quantitative evaluation of cell number was performed by ImageJ 1.50g (Java 1.8.0_181) software.

Human primary CD8+ T cell isolation and culture

The collection of peripheral blood from healthy donors was approved and performed at the Shanghai Cancer Center. The donor (female, 25 years old) consented to blood being used in the study. Blood samples were mixed with Ficoll Solution (Solarbio), and this mixture was centrifuged at 400× g with the lowest acceleration and deceleration speed for 25 minutes at room temperature. The peripheral blood mononuclear cells (PBMC) were carefully collected using a Pasteur pipette, centrifuged at 300×g for 10 minutes, and washed with PBS containing 2% FBS (Gibco) and 0.5% EDTA. After centrifugation, the PBMCs were incubated in ACK lysing buffer (NCM Biotech) for 5 minutes at room temperature to lyse RBCs and then neutralized by RPMI-1640 with 10% FBS and centrifuged at 300×g for 5 minutes. The PBMCs were then resuspended at 5E7 cells/mL and were isolated using the Human CD8+ T Cell Isolation Kit (BeaverBeads®), as per manufacturer's instructions. The isolated CD8+ T cells were cultured in X-VIVO® 15 Serum-free Hematopoietic Cell Medium (Bioscience) supplemented with 50 μM β-mercaptoethanol (MCE) at 1-1.5E6 cells/1-1.5mL, and incubated at 37 °C, 5% CO_2.

CD8+ T cell of tumor-killing assay

Human CD8+ T cells were briefly activated by 1μg/mL anti-CD3+ 0.5 μg/mL anti-CD28 antibodies in the presence of 100 IU/ mL rhIL2 for 24 hours. The activated CD8+ T cells were expanded for 3-4 days prior to co-culture. GFP-labeled MCF7 cells overexpressed MARCKSL1 or FKBP15 were seeded into a 96-well plate at a density of 2.5E3 cells/well. Following a 24-hour culture, the expanded CD8+ T cells were seeded into each well at different effector-to-target ratios in a complete T-cell medium

with 100 IU/ mL rhIL-2. The cells were imaged, and the GFP fluorescence of tumor cells was quantified for 72 hours, and tumor cells apoptosis was analyzed with LDH assay.

Quantification and Statistical Analysis

Proteome and Phosphoproteome Data Analysis and Quality Control

The protein intensity and phosphopeptide intensity obtained from the MaxQuant search result were used with Potential contaminant and Reverse removed. For phosphoproteome, the phosphopeptides with Localization probability less than 0.75 were excluded. The proteins/phosphopeptides with a total intensity of zero were also excluded. The intensities originating from the same protein/phosphorylation site were summed and then normalized by internal reference and sample median. The log2 transformed data was filtered to retain proteins with missing values in no more than 30% samples (50% for phosphorylation site). The missing values were then imputed using K-nearest neighbors (KNN) method.

Batch Effect and Quality Control for Proteomic and Phosphoproteomic Data

The batch effect was removed using ComBat from R package sva (v3.40.0). The principal component analysis (PCA) and Spearman correlation coefficient were used to assess the batch effect. For quality control, Spearman correlation coefficient analysis was performed on the internal reference. The log2 TMT ratios of the proteins or phosphorylation sites quantified in each sample were plotted as density plots, and a dip statistic test was employed to assess whether all samples passed the quality control with an expected unimodal distribution.

Differential Expression Analysis

The Wilcoxon signed-rank test was conducted to identify proteins, mRNA and phosphosites exhibiting significant differences between tumor and matched adjacent samples or between lymph node metastasis tumor samples and tumor samples. For comparisons among different groups of tumor samples (gene mutations vs wildtype, high expression vs low expression of protein/phosphorylation sites, different tumor stages, and different subtypes), the Wilcoxon rank-sum test was employed for identifying differentially expressed proteins. A significance criterion of p-values < 0.05 and a fold change of 1.2 were applied to proteome and phosphoproteome data and 1.5 were applied to transcriptome. To account for multiple testing, p-values were adjusted using the Benjamini-Hochberg FDR correction.

Bioinformatics Analysis

Pathway Enrichment Analysis

The pathway enrichment analysis was performed using both Over-Representation Analysis (ORA) and Gene Set Enrichment Analysis (GSEA) methods in R package ClusterProfiler (v4.12.0), or ClueGO (v2.5.10) in Cytoscape (v3.10.2). For ORA analysis, proteins or mRNAs showing significant expression changes (up-regulated or down-regulated) were used. For GSEA analysis, the ranked log2 fold of change was used. The ranked spearman correlation coefficients were used for correlation analysis in GSEA. The pathway databases were downloaded from MSigDB (v2022.1). Enrichment results with a false discovery rate (FDR) below 0.05 were considered statistically significant and visualized using either the R package or ggplot2.

Gene Set Variation Analysis (GSVA)

The imputed proteome or transcriptome data matrix was used as input for Gene Set Variation Analysis (GSVA) in the R (v4.1.0) package GSVA (v1.40.1). The pathway databases were downloaded from MSigDB (v2022.1).

Transcription Factor Activity Analysis

The transcription factor activity was calculated using single-sample gene set

enrichment analysis (ssGSEA) method in R package GSVA, and the database was downloaded from R package Dorothea (v1.4.2).

Kinase Activity Analysis

Kinase activity were analyzed using the following tools: Kinase-substrate enrichment analysis (KSEA), single-sample gene set enrichment analysis (ssGSEA), PTM Signature Enrichment Analysis (PTM-SEA).

The KSEA analysis was performed on the KESA App website (https://casecpb.shinyapps.io/ksea/) using phosphoproteome data and following its manual. The kinase-substrate dataset as "PhosphoSitePlus + NetworKIN" were selected. The ssGSEA (v1.0.0) analysis was performed in R package GSVA (v1.40.1) using phosphoproteome data. The Kinase-substrates information were downloaded from PhosphoSitePlus database (v6.7.1.1) and manually transform in to gmt format. The PTM-SEA were performed in R package ssGSEA2 using phosphoproteome data, and the pathway database was downloaded from PTM signatures database (PTMsigDB v2.0.0).

Correlation Analysis

The R package multiOmicsViz (version 4.3.1) was used to calculated and visualize the cis and trans effects of CNV-mRNA, CNV-Protein and CNV-Phosphoprotein. For gene-wise correlation analysis, the spearman correlation coefficient was employed to analyze mRNA-protein correlation in all samples, and the *p*-values were adjust using Benjamini-Hochberg adjustment methods. For sample-wise correlation analysis, spearman correlation coefficient was calculated for mRNA and protein quantification of same samples.

The correlation between protein and transcription factor activity, phosphosites and kinase activity, as well as metastasis lymph node number and protein intensity were analyzed using spearman correlation in R. The results were visualized using ggplot2.

Potential Therapeutic Targets Selection

The potential therapeutic targets were selected based on the following criteri: 1) Proteins had a generally expressed with missing values < 0.3 in all samples at proteome levels. 2) Proteins exhibited higher expression in lymph node metastasis tumors compared to tumor samples with fold of change > 1.2 and FDR < 0.05. 3) Proteins did not show specific expression in lymph nodes (download from the human protein atlas).

Survival Analysis

For survival analysis, the data downloaded from TCGA database were analyzed using R package survival (v3.5-8) and the cut point was determined using surv_cutpoint in R package survminer (v0.4.9). The Kaplan-Meier curve were plotted using ggsurvplot in R package survminer and other results were visualized using ggplot2.

RNA Splicing Analysis

The RNA splicing analysis was performed using rMATS (v4.1.2). The significant differences in splicing events were determined based on the absolute percent spliced in (PSI) difference > 0.1 and FDR < 0.1. The splicing results were visualized using rmats2sashimiplot.

Immune Analysis

For immune cell signature analysis, the xCell (v1.1.0) was used to estimate and calculate immune cell scores from transcriptome data (xCell and estimate v1.0.13).

Database Information

Proteins of enzyme, FDA approved drug targets, potential drug targets, protein subcellular localization and secreted proteins were downloaded from the human protein atlas database.

Statistical Analysis

Statistical analysis was performed using Wilcoxon signed-rank test with paired for tumor and normal, tumor and lymph node metastasis tumor, lymph node metastasis tumor and normal. *p*-values were adjusted using Benjamini-Hochberg method. All correlation analysis were performed using spearman correlation method. *P* values or adjusted *p* values < 0.05 were considered statistically significant. The sample sizes were determined by selecting all available samples that met inclusion and exclusion criteria.
